# Does Industry-Driven Alcohol Marketing Influence Adolescent Drinking Behaviour? A Systematic Review

**DOI:** 10.1093/alcalc/agw085

**Published:** 2016-12-20

**Authors:** Stephanie Scott, Colin Muirhead, Janet Shucksmith, Rachel Tyrrell, Eileen Kaner

**Affiliations:** 1 Institute of Health & Society, Baddiley-Clark Building, Newcastle University, Richardson Road, Newcastle upon Tyne NE2 4AX, UK; 2 Health and Social Care Institute, Parkside West Offices, Teesside University, Middlesbrough, Tees ValleyTS1 3BA, UK; 3 Centre for Early Child Development, NSPCC, Number One, Bickerstaffe Square, Blackpool FY1 3AH, UK

## Abstract

**Aim:**

To systematically review evidence on the influence of specific marketing components (Price, Promotion, Product attributes and Place of sale/availability) on key drinking outcomes (initiation, continuation, frequency and intensity) in young people aged 9–17.

**Methods:**

MEDLINE, EMBASE, SCOPUS, PsychINFO, CINAHL and ProQuest were searched from inception to July 2015, supplemented with searches of Google Scholar, hand searches of key journals and backward and forward citation searches of reference lists of identified papers.

**Results:**

Forty-eight papers covering 35 unique studies met inclusion criteria. Authors tended to report that greater exposure to alcohol marketing impacted on drinking initiation, continuation, frequency and intensity during adolescence. Nevertheless, 23 (66%) studies reported null results or negative associations, often in combination with positive associations, resulting in mixed findings within and across studies. Heterogeneity in study design, content and outcomes prevented estimation of effect sizes or exploration of variation between countries or age subgroups. The strength of the evidence base differed according to type of marketing exposure and drinking outcome studied, with support for an association between alcohol promotion (mainly advertising) and drinking outcomes in adolescence, whilst only two studies examined the relationship between alcohol price and the drinking behaviour of those under the age of 18.

**Conclusion:**

Despite the volume of work, evidence is inconclusive in all four areas of marketing but strongest for promotional activity. Future research with standardized measures is needed to build on this work and better inform interventions and policy responses.

## INTRODUCTION

Heavy drinking or high intensity alcohol use is the leading risk to health and well-being in young people, accounting for 7% of disability adjusted life years in 10–24-year olds globally (Gore *et al*., 2011). Short-term implications, which pose the greater immediate threat to health and well-being, include accidents, violence and assaults, early and unprotected sex, exacerbation of mental health problems, and poor school attendance and educational attainment (Witt, 2010). Acute problems may have lifetime consequences such as permanent disfigurement or unintended pregnancies. Early onset or initiation of alcohol use has been reported to be a strong predictor of alcohol problems and dependence in adulthood (Grant *et al*., 2001; Bonomo *et al*., 2004; Kuntsche *et al*., 2013), although a recent systematic review has challenged this finding, highlighting a lack of concrete and direct longitudinal data or evidence of tracking (Maimaris and McCambridge, 2013). Nevertheless, the longer and heavier an individual drinks, the greater the risk of developing chronic health problems such as liver disease or cancers later in life (Secretan *et al*., 2009; Holmes *et al*., 2014b; Williams *et al*., 2014; Bagnardi *et al*., 2015).

The impact of alcohol marketing on consumption has received considerable attention, both in terms of research and public policy, with the World Health Organisation (WHO) identifying the regulation of alcohol marketing as one of three ‘best buy’ policies for reducing harms in its Global Alcohol Strategy (World Health Organisation, 2010), alongside restrictions on availability and affordability. In reality, the marketing of alcohol is a complex process, comprising four well-established and interconnected domains: pricing, product launch and development (characteristics, image and branding), promotional activity (including both above and below the line advertising) and placement (point of sale marketing or distribution) (Hastings *et al*., 2005; Sheron and Gilmore, 2016). A growing body of literature, including two systematic reviews, has reported an association between exposure to aspects of alcohol marketing and initiation or progression (continued use) of alcohol use among young people (Anderson *et al*., 2009; Smith and Foxcroft, 2009). However, as the associated literature searches went up to 2008 and 2006, respectively, these reviews were unable to examine emergent marketing strategies, including digital marketing. Alcohol marketing communications are increasingly present in diverse forms, from traditional television advertisements to ‘new media’ platforms such as social network sites, viral campaigns and the sponsorship of products, services and events. Indeed, European alcohol advertising on the internet (post 2009) comprised one-fifth of the advertising expenditure of all media measured (internet, television, magazines and newspapers) (EACA, 2009). More recently, a third systematic review of longitudinal studies published since 2008, updating the systematic reviews conducted by Smith and Foxcroft (2009) and Anderson *et al*. (2009), concluded that young people who have greater exposure to alcohol marketing appear to be more likely to subsequently initiate alcohol use and engage in binge and hazardous drinking (Jernigan *et al*., 2016).

Nevertheless, all three reviews considered only one aspect of marketing (promotion). Four further systematic reviews have considered marketing influences, including placement and availability (Meier *et al*., 2008; Bryden *et al*., 2012), price (Meier *et al*., 2008), advertising (Meier *et al*., 2008; Bryden *et al*., 2012; Stautz *et al*., 2016) and sports sponsorship (Brown, 2016) on alcohol consumption. Meanwhile, a recent systematic review has investigated the content of, and exposure to, alcohol marketing in relation to self-regulated guidelines (Noel *et al*., 2016). However, these reviews included both adults and adolescents. One final systematic review has explored the relationship between exposure to internet-based alcohol content and alcohol use among young people (Gupta *et al*., 2016). Yet, exposure measures of interest in this review were both user-generated and industry-led. Therefore, our study aimed to systematically review the evidence on the influence of specific alcohol marketing (Price, Promotion, Product and Placement) on behavioural drinking outcomes (initiation, continuation, frequency and intensity) for adolescents only (young people aged 9–17).

## METHODS

### Eligibility criteria

Primary studies of any design, published in the English language, which examined the relationship between marketing exposure and alcohol consumption outcomes among 9–17 (inclusive)-year olds were eligible for inclusion. Papers which also included participants aged 18 or more were excluded if data on adolescents were not presented separately. The exposure of interest was industry-led alcohol marketing. We defined this to include the following practices: (a) price promotions, discounts or changes, (b) promotion in terms of any measurable exposure to alcohol products/images or associated merchandise, (c) product launch and development (including characteristics, image and branding) and (d) placement point of sale marketing or distribution, including reported density of off-premise outlets (e.g. shops) and on-premise outlets (e.g. bars). We defined the ‘alcohol industry’ as any company that produces, markets or distributes alcoholic beverages (Brown, 2016). Thus, we excluded studies that focused upon aspects of alcohol availability (such as server behaviour, parental supply of alcohol) or policy-level intervention (such as minimum age laws, restrictions on hours, days and volumes of alcohol sales in a given community) not driven by industry. Studies reporting general media alcohol portrayal (e.g. seeing actors drinking alcohol in a TV show), product placement or where participants were presented with alcohol imagery and assessed in an artificial environment were also excluded from this review, as were studies reporting proxy outcome measures (e.g. alcohol-related hospital admissions; purchasing behaviour) or drinking intentions only without measuring effects on consumption. Whilst product placement is considered to be predominantly industry-led, it is difficult to disentangle this practice from general portrayal of alcohol products, resulting in little available data on drinking impact.

### Search strategy

One author (S.S.) searched MEDLINE, EMBASE, SCOPUS, PsychINFO, CINAHL and ProQuest databases (including CSA Illumina) from inception to July 2015 using appropriate MeSH terms. The search was split into three core concepts—(a) alcohol consumption (initiation, continuation, frequency and intensity), (b) participants (young people aged 9–17) and (c) marketing techniques (full details of database-specific search terms available upon request from the corresponding author). Database searching was supplemented with searches of Google Scholar, hand searches of key journals and backward and forward citation searches of reference lists of identified papers. Key journals were defined as the most common five journals revealed by electronic searches. Relevant websites and grey literature (including theses, conference abstracts, unpublished/ongoing studies and reports) were also examined.

### Study selection and data extraction

The title and abstract of all records retrieved were downloaded to Endnote X7 and independently screened by two reviewers (S.S. and R.T.), with full text copies of potentially relevant papers retrieved for in-depth review against the inclusion criteria. Uncertainties were resolved through discussion and referral to a third member of the review team (E.K.). Data were independently extracted by two authors (S.S. and R.T.) on study design; individuals’ exposure to alcohol marketing; alcohol drinking behaviour; characteristics of the sample population; study results and author conclusions; limitations of the study; reported analyses and analysis type. Primary outcomes of interest were reported changes in participants’ behaviour in relation to drinking initiation, continuation, frequency and intensity measures. Odds ratios (OR), adjusted odds ratios (AOR), 95% confidence intervals (CI) and other measures reported were extracted. We defined mixed findings as any combination of positive associations, negative associations and null results. Negative associations were defined as significant decreases in drinking linked to alcohol marketing, whereas null results were defined as no reported effect of alcohol marketing on drinking behaviour.

### Data synthesis and assessment of methodological quality

The methodological quality of all studies was assessed independently by two researchers (S.S. and R.T.) as strong, moderate or weak using the Effective Public Health Practice Project (EPHPP) Quality Assessment Tool (National Collaborating Centre for Methods and Tools, 1998); studies were not excluded on the basis of the overall quality rating. The aim of this review was to systematically identify and synthesize the full evidence base. Quality assessment supported data synthesis by providing an indication of the degree of confidence that could be placed on findings from different studies based on their risk of bias. Studies were combined using narrative synthesis, structured according to drinking outcome of interest (e.g. initiation) followed by marketing technique (e.g. price). Heterogeneity in terms of study designs, study populations and the exposure/outcomes measured precluded the use of meta-analytical techniques. Reporting adhered to PRISMA statement guidelines (Moher *et al*., 2009).

## RESULTS

### Description of included studies

**Fig. 1.**
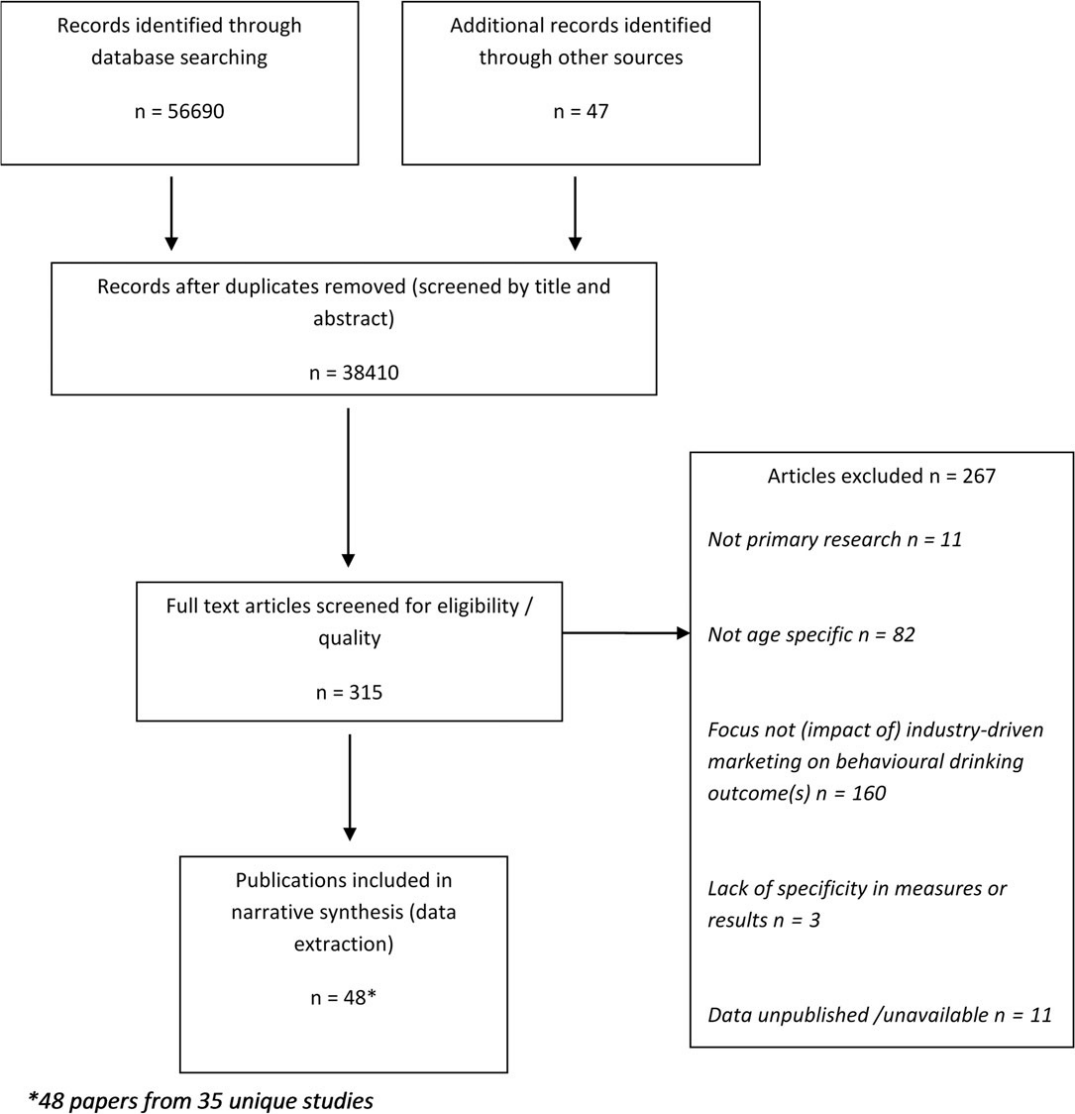
Flow chart showing study selection process.

The review identified 48 publications covering 35 unique or ‘index’ studies (see Fig. 1). Analysis focused on the latter (but flagged linked references) to avoid placing undue weight on specific evaluations due to multiple reports. Participants ranged between 9 and 17 years old at baseline; sample sizes ranged from 172 to 371,194 participants. In total, 57% (*n* = 20) of the studies originated in the USA (Workman, 2003; Stacy *et al*., 2004; Zogg, 2004; Ellickson *et al*., 2005; McClure *et al*., 2006; Saffer and Dave, 2006; Collins *et al*., 2007; Fisher *et al*., 2007; Paschall *et al*., 2007; Dumsha, 2008; Grenard, 2008; Henriksen *et al*., 2008; Truong, 2008; McClure *et al*., 2009; Pasch *et al*., 2009; Tobler, 2009; Tobler *et al*., 2009; Truong and Sturm, 2009; Chen *et al*., 2010; Dumsha, 2011; Reboussin *et al*., 2011; Shamblen *et al*., 2011; Stanley *et al*., 2011; Tobler *et al*., 2011; Stoolmiller *et al*., 2012; Grenard *et al*., 2013; Lo *et al*., 2013a, b; McClure *et al*., 2013; Rowland *et al*., 2014), 6% (*n* = 2) from the UK (Gordon *et al*., 2010a, b; Gordon, 2011; Young *et al*., 2013), 9% (*n* = 3) from Australia (Jones and Magee, 2011; Rowland *et al*., 2014; Azar *et al*., 2016), 6% (*n* = 2) from New Zealand (Huckle *et al*., 2008; Lin *et al*., 2012) and one study from Denmark (Bendtsen *et al*., 2013), Brazil (Faria *et al*., 2011), Switzerland (Kuntsche *et al*., 2008), Zambia (Swahn *et al*., 2011), the Philippines (Swahn *et al*., 2013) and the Netherlands (van Hoof *et al*., 2008). Two further studies (6%) spanned several European countries, with data collected in Germany, Italy, Scotland, the Netherlands and Poland (de Bruijn *et al*., 2012; Anderson *et al*., 2012; Morgenstern *et al*., 2014; de Bruijn *et al*., 2016a, b). The majority of studies focused exclusively on alcohol promotion (*n *= 14, 40%) or its placement in retail outlets (*n* = 13, 37%), with a much smaller number examining price (*n* = 1) or the development, launch or branding of alcohol products (*n* = 1) only. The remaining studies cut across more than one area of marketing (*n* = 6). Categorization of studies according to marketing focus was guided by criteria from the UK's National Institute for Health and Care Excellence (NICE) (2010) and the WHO (World Health Organisation, 2010). A summary of included studies, including exposure and behavioural outcome measures, as well as participant characteristics (age, gender, ethnicity and socio-economic status where reported) is provided in the Supplementary material (Supplementary Table S1). The following sections and Supplementary Table S2 report findings first by methodological quality and then by key drinking outcomes (initiation, continuation, frequency and intensity) and marketing technique (Price, Placement, Product and Promotion). Finally, Supplementary Table S3 reports specifically on the relationship between alcohol promotions and binge drinking. Specific negative associations and null results are shaded grey in Supplementary Tables S2 and S3.

### Methodological quality

Our assessment found that 46% (*n* = 16) of included studies were categorized as having a high risk of bias whilst 23% (*n* = 8) were categorized as methodologically strong. Most papers presented cross-sectional data, with a predominance of self-reported rather than objective exposure and outcome measures, precluding the possibility of causal attribution. One study reported findings from a quasi-experiment (Dumsha, 2008) whilst a third of studies (*n* = 12) were longitudinal in design, seven of which (58%) were categorized as methodologically ‘strong’, and better able to examine the impact of alcohol marketing from early to later adolescence and beyond (Stacy *et al*., 2004; McClure *et al*., 2006; Fisher *et al*., 2007; Tobler, 2009; Shamblen *et al*., 2011; de Bruijn *et al*., 2012; Morgenstern *et al*., 2014). Additional criteria drew these seven longitudinal and methodologically strong studies together. Six focused on alcohol promotional activity, with three studies specifically exploring the effect on reported consumption of owning alcohol merchandise. Outcome measures relating to drinking initiation and drinking volume also featured heavily in this subset of studies (five studies). Outcome measures reported in six (of seven) studies were dichotomized. Nevertheless, outcome measures were not standardized (and could not be combined statistically); and these longitudinal studies continued to demonstrate heterogeneity in target behaviours and exposure measures.

### Drinking initiation (10 studies; 4 methodologically strong)

Price: no studies

Placement: no studies

*Product: n = *1

Dumsha (2008) reported null results and found the introduction of alcopops to have no immediate or long-term effect on age at first drink among US adolescents aged 14–17; study results did not differ when stratified by age, gender or ethnicity.

*Promotion: n = *9

All study authors reported that—to some degree—alcohol promotion influenced drinking initiation among young people. Nevertheless, exposure and outcome measures varied considerably across studies (see Supplementary Table S1). Four studies (of 9, 44%) were categorized methodologically ‘strong’ (McClure *et al*., 2006; Fisher *et al*., 2007; Gordon, 2011; Morgenstern *et al*., 2014). Furthermore, of this grouping of studies, one was longitudinal in design, and demonstrated that owning and/or being willing to use alcohol merchandise was associated with higher drinking uptake, particularly among older (aged 15 or over) rather than younger US boys (older: OR = 2.43, CI = 1.51–3.91; younger: OR = 1.50, CI = 1.08–2.09, *P* = 0.08) (Fisher *et al*., 2007). Three studies reported both positive associations and null results (Ellickson *et al*., 2005; Gordon, 2011; Jones and Magee, 2011). Thus, Ellickson *et al*. (2005) reported that exposure to in-store alcohol displays was the only measure (of four measures studied) of alcohol promotion that significantly predicted drinking initiation at follow-up in US adolescents aged 13–15 (OR = 1.42; *P*< 0.05). Gordon (2011) found that, whilst participation in alcohol marketing at baseline significantly increased the odds of drinking initiation at follow-up among Scottish 14–16-year olds (AOR = 1.31, CI = 1.003–1.711, *P* < 0.05), the number of brands recalled at baseline had no significant impact. Meanwhile, Jones and Magee (2011) reported that the relationship between alcohol promotion and drinking initiation varied by age and gender, finding no significant association between exposure to alcohol promotional media (of any type studied) and drinking initiation for Australian males and females aged 12–15 and males aged 16–17.

### Drinking continuation (12 studies; 3 methodologically strong)

*Price: n = *1

van Hoof *et al*. (2008) found that alcohol discounts had a significant effect on alcohol consumption among young people aged 14–17 in the Netherlands (*m* = 3.39, SD = 0.76, *t*[149] = 6.25, *P* = 0.000). This effect did not significantly differ between age groups (14–15 and 16–17) (*t*[138,54] = 0.91, *P* = 0.367).

*Placement: n = *3

Whilst all study authors identified that—to some degree—alcohol placement influenced continued alcohol use among young people, all three studies reported both positive associations and null results. Two (of three) studies were categorized methodologically ‘strong’ (Tobler, 2009; Shamblen *et al*., 2011). Shamblen *et al*. (2011) found that US students in high off-trade outlet density communities increased their alcohol use between sixth and eighth grades (equivalent age range: 11–14); but that students attending schools in low outlet density communities had higher initial levels of alcohol use that remained relatively stable over time (lifetime: OR = 0.87; past year: OR = 0.88; past 30-days: OR = 0.88). Furthermore, Tobler reported that outlet density did not have a significant direct effect on alcohol use in eighth grade. Furthermore, among low-income US African-American adolescents, the effects of alcohol outlet density at baseline on alcohol use in 12th grade were mediated entirely by beliefs favourable to use (equivalent age: 17–18 years) (*β* = 0.037, *P* = 0.001) and deviant peer affiliations (*β* = 0.016, *P* = 0.017). Meanwhile, Rowland *et al*. (2014) identified that all types of outlet density (general density, packaged outlet density, on-premise density and club density) were associated with increased alcohol use, but only when included as an interaction with age. For every 10% increase in overall alcohol density outlet, a significant percentage increase (PI) in adolescent alcohol consumption occurred for Australian young people aged 12–14 years (12: PI = 2.04, CI = 0.74–3.35; 13: PI = 1.66, CI = 0.53–2.80; 14: PI = 1.12, CI = 0.14–2.11) but not for those aged 15–17 (15: PI = 0.29, CI = −7.32–1.31; 16: PI = −1.19, CI = −2.91–5.29; 17: PI = −4.452, CI = −9.04–0.01).

*Product: n = *3

Lin *et al*. (2012) found that having a favourite alcohol brand increased the odds of being a drinker among 12–15-year olds in New Zealand (OR = 4.56, CI = 3.62–5.76); whilst Henriksen *et al*. (2008) identified that small increases in the odds of alcohol use at follow-up were associated with better brand recall at baseline among US 10–15-year olds (brand recall: OR = 1.13, CI = 0.94–1.33). However, as with drinking initiation, Dumsha (2008) reported null results and found the introduction of alcopops had no immediate or long-term effect on drinking continuation among US adolescents aged 14–17; study results did not differ when stratified by age, gender or ethnicity.

*Promotion: n = *8

Two studies (of 8, 25%) were categorized methodologically ‘strong’ (Stacy *et al*., 2004; Tobler, 2009). However, findings from both studies were not clear-cut, with both positive associations and null results reported. Stacy *et al*. (2004) identified that seventh-grade exposure to advertising via popular TV shows (equivalent age: 12–13 years) was the only measure (of three measures studied) of alcohol promotion that was significantly associated with eighth-grade beer and wine/liquor use among US young people (equivalent age: 13–14 years) (AOR = 1.44, CI = 1.27–1.61, *P* < 0.001). Furthermore, Tobler (2009) demonstrated that exposure to alcohol advertisements was significantly associated with alcohol use in eighth grade (*β* = 0.049, *P* < 0.05). However, among US low-income Hispanic adolescents, the effects of alcohol advertisement exposure on alcohol use in 12th grade (equivalent age: 17–18 years) were entirely mediated through beliefs favourable to use in 8th grade (*β* = 0.014, *P*= 0.076). Whilst exclusively positive associations were reported in three studies (Collins *et al*., 2007; Henriksen *et al*., 2008; Lin *et al*., 2012), negative associations and null results were also reported by Swahn *et al*. (2011) and Zogg (2004). Thus, despite reporting a positive relationship between provision of free alcohol from an industry representative and current alcohol use (OR = 4.37, CI = 3.21–5.95), Swahn *et al*. (2011) found exposure to billboard advertisements was associated with ‘decreased’ reported alcohol use among Zambian young people aged 11–16 (OR = 0.65, CI = 0.46–0.92). Meanwhile, Zogg (2004) identified that exposure to TV alcohol advertisements during the seventh grade significantly predicted beer use 1 year later (*P* < 0.05), but not wine or liquor use. Furthermore, Zogg (2004) also found that exposure to alcohol advertising around popular TV shows and TV sports in seventh-grade predicted beer and wine or liquor use by eighth grade, but only for white respondents (TV shows: *P*< 0.001; wine/liquor: *P* < 0.001; TV sports: beer: *P* < 0.01; wine/liquor: *P* < 0.05).

### Drinking frequency (19 studies; 2 methodologically strong)

Price: no studies

*Placement:* *n = *10

Paschall *et al*. (2007) measured school district-level alcohol sales across 3 months whilst nine studies focused upon community-level outlet density (Huckle *et al*., 2008; Kuntsche *et al*., 2008; Truong, 2008; Pasch *et al*., 2009; Chen *et al*., 2010; Stanley *et al*., 2011; Lo *et al*., 2013a; Young *et al*., 2013; Azar *et al*., 2016). Young *et al*. (2013) reported significant associations between the proximity and density of alcohol off-sales outlets and weekly drinking among Scottish 15-year olds; and Kuntsche *et al*. (2008) identified that on-premise outlet density was positively related to quantity frequency drinking (no. of drinks consumed in a typical occasion multiplied by frequency of alcohol use) among 12–17-year olds in Switzerland (*P* < 0.05). Nevertheless, one study reported a mixture of positive associations and null results (Azar *et al*., 2016); two studies identified negative as well as positive associations (Chen *et al*., 2010; Stanley *et al*., 2011); while four studies found no relationship whatsoever between alcohol outlet density and drinking frequency (Huckle *et al*., 2008; Truong, 2008; Pasch *et al*., 2009; Lo *et al*., 2013a, b) (see Supplementary Table S2). Furthermore, despite a domination of studies measuring alcohol placement and drinking frequency, none were categorized methodologically ‘strong’ (moderate: *n* = 5; weak: *n* = 5).

*Product:* *n = *2

Faria *et al*. (2011) found past 30-day drinking to be associated with having a favourite alcohol brand among Brazilian 11–16-year olds (OR = 5.150, CI = 3.355–7.906, *P* < 0.001); whilst Lin *et al*. (2012) identified that having a favourite alcohol brand increased frequency of alcohol consumption among 12–15-year olds in New Zealand (OR = 1.65, CI = 1.41–1.92).

*Promotion: n = *9

All study authors identified that—to some degree—alcohol promotion influenced drinking frequency among young people. Five (of 9, 56%) studies reported wholly positive associations, whilst three reported null results and one identified a negative association, in addition to positive associations. One study was categorized methodologically ‘strong’ (Gordon, 2011) yet reported null results as well as positive associations between alcohol promotion and drinking frequency. Specifically, the authors found that higher marketing involvement at baseline significantly increased the odds of fortnightly and monthly drinking at follow-up among Scottish 14–16-year olds (fortnight: AOR = 1.43, CI = 1.146–1.795, *P* < 0.01; monthly: AOR = 1.33, CI = 1.072–1.644, *P* < 0.05). Uptake of fortnightly drinking at follow-up was also significantly associated with marketing awareness at baseline (AOR = 1.11, CI = 1.005–1.234, *P*< 0.05). However, they found no association between uptake of fortnightly drinking at follow-up and number of brands recalled at baseline; and no association between uptake of monthly drinking at follow-up and awareness of alcohol marketing or number of brands recalled at baseline. Three studies found a positive relationship between exposure to alcohol marketing via the internet or social media platforms and drinking frequency (Jones and Magee, 2011; de Bruijn *et al*., 2012; Lin *et al*., 2012). Thus, de Bruijn *et al*. (2012), categorized as methodologically ‘strong’, found that the extent of exposure to online alcohol marketing among European 14-year olds was associated with frequency of past 30-day alcohol use 14–15 months later (*P* < 0.001). Furthermore, Jones and Magee (2011) reported exposure to advertising via the internet predicted past 4-week alcohol use among Australian 12–17-year olds (AOR = 1.36, CI = 1.03–1.79); and Lin *et al*. (2012) found that engagement with both traditional and web-based alcohol marketing increased frequency of alcohol consumption among 12–15-year olds in New Zealand (OR = 1.34, CI = 1.08–1.66).

### Drinking intensity (19 studies; 4 methodologically strong)

*Price: n = *1

Using two large data sets, Saffer and Dave (2006) demonstrated that binge drinking among US adolescents (mean age: 15 years) reduced as price increased (data set 1 (MTF): price elasticity: −0.1842, SE = 0.0562; data set 2 (NLSY): price elasticity: −0.7307, SE = 0.4897). Effects were larger for females and white young people (data set 1 (MTF): female: −0.2369, SE = 0.0803; white: −0.3611, SE = 0.0658).

*Placement: n =* 8

No studies that measured alcohol placement were categorized as methodologically ‘strong’ for high intensity drinking (moderate: *n* = 4; weak: *n* = 4). Two studies (of 8, 25%) found no relationship between alcohol placement and drinking intensity (Pasch *et al*., 2009; Bendtsen *et al*., 2013). Furthermore, no studies reported wholly positive associations. Thus, 4 studies (of 8, 50%) identified both positive associations and null results (Huckle *et al*., 2008; Kuntsche *et al*., 2008; Truong, 2008; Azar *et al*., 2016), whilst 2 studies (of 8, 25%) reported both positive and negative associations (Chen *et al*., 2010; Lo *et al*., 2013a). Specifically, Lo *et al*. (2013a) found that alcohol outlet density was significantly associated with ‘reduced’ binge drinking in the past 2 weeks (5 or more drinks in a 2-hour time period) among US 11–18-year olds (*P* < 0.05), but that binge drinking increased with grade level, and that this association became stronger among students living in neighbourhoods with high alcohol outlet density (*P* < 0.05). Meanwhile, Chen *et al*. (2010) identified that, among US 14–16-year olds, the initial level of frequency of excessive drinking (defined as number of days drunk in the past 12 months) related positively to outlet density (coefficient = 0.0009, *P* = 0.000). Nevertheless, growth of frequency of excessive drinking was related negatively to outlet density (coefficient = 0.0004, *P*= 0.008). Furthermore, the relationship between outlet density and drinking was mitigated by friends with access to a car.

*Product: n = *2

As with both drinking initiation and continuation, Dumsha (2008) reported null results and found the introduction of alcopops had no immediate or long-term effect on episodic heavy drinking among US adolescents aged 14–17; study results did not differ when stratified by age, gender or ethnicity. However, reporting similar results to that which they found for drinking continuation and frequency, Lin *et al*. (2012) identified that having a favourite alcohol brand increased drinking amount on a typical drinking occasion (OR = 1.86, CI = 1.57–2.21) among 12–15-year olds in New Zealand.

*Promotion: n = *10

Four studies (of 10, 40%) were categorized methodologically ‘strong’ (Stacy *et al*., 2004; Fisher *et al*., 2007; de Bruijn *et al*., 2012; Morgenstern *et al*., 2014). All four studies were longitudinal in design. Thus, Morgenstern *et al*. (2014) reported that having a favourite advertisement was significantly associated with binge drinking among young people in Germany, Italy, Poland and Scotland (mean age: 13.5 years old) (AOR = 2.13 CI = 1.92–2.36). Studies which addressed drinking intensity or ‘binge’ had the greatest degree of consistency in terms of exposure measures and a common outcome. Across the entire review, 12 studies reported binge drinking (measured as 5 or more drinks in a specified time period) as the outcome measure. More specifically, seven of these studies considered the relationship between alcohol promotions and binge drinking (see Supplementary Table S3). Reported exposure to alcohol promotions in five of these studies fell into two groups: (a) self-reported recall of adverts as a scale (ordinal) measure (Stacy *et al*., 2004; Zogg, 2004; de Bruijn *et al*., 2012) and (b) ownership and/or awareness of alcohol merchandise as a binary (yes/no) measure (Fisher *et al*., 2007; McClure *et al*., 2009; de Bruijn *et al*., 2012). Four (of 7, 57%) promotional studies reported only positive associations with binge drinking (Saffer and Dave, 2006; McClure *et al*., 2009; de Bruijn *et al*., 2012; Morgenstern *et al*., 2014), two reported mixed results (positive associations and null results) (Zogg, 2004; Fisher *et al*., 2007), whilst one reported no relationship (Stacy *et al*., 2004). Thus, Fisher *et al*. (2007) found that owning and/or being willing to use alcohol merchandise predicted binge drinking 12 months later for US girls aged 11–18 but not boys (girls: OR = 1.79, CI = 1.16–2.77; boys: OR = 0.87, CI = 0.51–1.48); and Zogg (2004) reported that self-reported exposure to TV alcohol advertisements in seventh grade (equivalent age: 12–13 years) did not predict binge drinking for US adolescents by eighth grade (equivalent age: 13–14 years). Furthermore, exposure to alcohol advertising around popular TV shows and TV sports in seventh grade significantly predicted binge drinking by eighth grade for white respondents only (TV shows: *P* < 0.05; TV sports: *P* < 0.01). Meanwhile, Stacy *et al*. (2004) identified that eighth-grade binge drinking among US adolescents was not significantly associated with any measure of seventh-grade advertising exposure studied (self-reported exposure to TV alcohol advertising, exposure to advertising around popular TV shows and exposure to advertising around TV sports).

## DISCUSSION

This review found a diverse literature spanning many countries though dominated by the USA. Twelve (of 35, 34%) studies reported wholly positive associations; 20 (57%) reported mixed findings (combinations of positive, negative and null results) and 3 (9%) reported no relationship between alcohol marketing and alcohol use among young people aged 9–17. The strength of the evidence differed according to the type of marketing exposure and drinking outcome studied. Only two studies identified by this review focused on alcohol price and drinking behaviour in those under the age of 18. Whilst both studies reported only positive associations, both of these studies were categorized as methodologically ‘weak’, and price and affordability remains a significantly understudied influence upon young people's drinking behaviour. Previous authors have reported that price and affordability might not be as effective at reducing drinking in young people as in adults, or as compared to other strategies such as point of sale or offer restrictions (Meier *et al*., 2009). As work in Scotland has concluded, young adults are not a homogeneous group in relation to price sensitivity, and considerations about the price of alcohol compete with non-financial considerations such as cultural norms regarding drinking activity (Seaman *et al*., 2013). Eleven (of 14) placement studies reported mixed results. A further two reported null results only. Furthermore, whilst alcohol placement is not limited to outlet density *per se*, published studies in this area tended to focus their attention here (or upon similar measures such as outlet proximity). We were interested in studies which specifically reported effects on young people's (aged 9–17 years) alcohol consumption, and found very little which focused upon other aspects of placement or distribution, reflecting methodological gaps and limitations previously identified by Holmes *et al*. (2014a). We also identified few data which took into account targeted geographical positioning of alcohol outlets (and neighbourhood deprivation) when reporting the association between alcohol placement and objective consumption measures.

The largest cluster of evidence uncovered (*n* = 20) points to a relatively consistent association between alcohol promotion (predominantly advertising) and drinking outcomes in adolescence. Ten studies (of 20, 50%) reported exclusively positive associations and this relationship is supported by wider literature; previous research indicates that alcohol brand recognition occurs in 10–11-year olds (Alcohol Concern, 2012), while identification with desirable images in alcohol advertising has been seen in 8–9-year olds and brand-specific consumption in 13–20-year olds (Austin *et al*., 2006; Siegal *et al*., 2013). However, establishing causality between promotional activity and alcohol use is methodologically and ethically problematic, especially where subjects are under the legal age for purchasing alcohol. This is compounded further by the difficulty in separating advertisements aimed at adults from those aimed at children and young people, and the growth of marketing in youth focused outlets, in formats that are likely to appeal to children and young people (Hastings *et al*., 2010; Barry *et al*., 2016).

In psychological theory, exposure to marketing is thought to stimulate a motivation to drink alcohol via both conscious (explicit) and non-conscious (implicit) processes (Stautz *et al*., 2016). Conscious processes include increasing positive expectancies and making attitudes more favourable; non-conscious processes include imitation, modelling and priming. Thus, positive alcohol-related cognitions may be activated immediately in response to a single exposure, as well as develop over time in response to repeated exposures (Stautz *et al*., 2016). However, this framework assumes a linear ‘effect’ where marketing activity acts like a ‘hypodermic syringe’, injecting passive viewers with information which creates attitudes and behaviours in response (Baillie, 1996). In reality, individuals have the capacity to accept, reconstruct or reject the information they receive (Atkinson *et al*., 2013) and may interpret marketing messages differently (Scott *et al*., 2014). Work by Morgenstern *et al*. (2011) highlights the importance of attitudes as mediators of behaviour using the message interpretation process model, which suggests that the effectiveness of any marketing message is dependent on the formation of alcohol-related expectancies, namely desirability, identification and scepticism (Austin and Knaus, 2000; Austin *et al*., 2006).

Increasingly, marketing is understood to operate within a rich milieu of other social and cultural influences on behaviour: behavioural drivers may work together to ‘collectively’ influence young people's drinking practices. This corroborates established theories of social behaviour which argue for the interaction between individual agency and social structure (Cockerham, 2005), e.g. Bourdieu's theory of practice (Bourdieu, 1990), aspects of which have been applied to the study of young people's alcohol use (Jarvinen and Gundelach, 2007; Lunnay *et al*., 2011). Bourdieu's framework rests upon three core concepts. ‘Habitus’ representing an embodied yet flexible system of shared tastes, habits and dispositions (Brierley-Jones *et al*., 2014); ‘field’, a person's position in social, physical and digital space—those who occupy a proximal position often share similar lifestyles; and the type and amount of ‘capital’ (economic, cultural, social or symbolic) or assets an individual possesses relative to others (Demant and Jarvinen, 2011; Browne-Yung *et al*., 2013; Christensen and Carpiano, 2014). Together, these concepts generate ‘practices’. Applied to drinking practices, extensive, often subliminal, marketing lead to it becoming a seemingly ordinary and often subconscious aspect of daily life (Hastings *et al*., 2010), creating an ‘intoxigenic’ environment where social, physical and regulatory influences shape youth drinking (McCreanor *et al*., 2008; Townshend, 2013). Marketers can reinforce aspects of the surrounding social ecology, by encouraging a link between alcohol and aspects of culture, identity and personal reward (Brierley-Jones *et al*., 2014). The drinks industry works to develop an ongoing multifactorial relationship with consumers rather than aiming for a straightforward transaction (Nicholls, 2012). This relationship may begin earlier than previously assumed, being well under way in some young people by mid-adolescence (Scott *et al*., 2014). Such relationships may be subtle and gradual, unlikely to be observed in studies investigating only the immediate effects of marketing exposure (Stautz *et al*., 2016). In addition to industry-led marketing messages, recent studies have identified associations between assuming an ‘alcohol identity’ online and harmful drinking behaviour (Ridout *et al*., 2012), illustrating a blurring of boundaries between commercial advertising and user-generated content (Moreno *et al*., 2012).

## STRENGTHS AND LIMITATIONS

Studies varied in terms of exposure type and measures as well as reported behavioural outcomes. This heterogeneity made it difficult to synthesize key findings, or to explore variation between countries or age subgroups and prevented quantitative meta-analysis for estimating effect sizes. The quality of statistical reporting sometimes made it difficult to interpret findings. We extracted beta, standard error and coefficient values as reported in published papers. However, at times, the meaning of these values was not clear. Furthermore, a different terminology was used to describe drinking behaviour across identified studies. For example, whilst most referred to high intensity drinking as ‘binge’ drinking, others described comparable behaviour as ‘drunkenness’, ‘episodic’, ‘risky single occasion’, ‘heavy’ or ‘excessive’ drinking. Where used, ‘binge’ drinking was typically measured as 5+ drinks in a single occasion/within a couple of hours. Nevertheless, the time frame varied from within the last fortnight to as much as a month or year. Most studies reported ethnic background, race or migration status as part of sample characteristics, many of which identified associations between ethnicity and alcohol use. Nevertheless, only six studies (Zogg, 2004; Saffer and Dave, 2006; Dumsha, 2008; Tobler, 2009; Chen *et al*., 2010; Stanley *et al*., 2011) reported multivariate analysis of associations between ethnic background, marketing exposure and alcohol use (see Supplementary Table S2). Thus, whilst there is evidence to demonstrate that adolescents from different ethnic backgrounds respond to marketing differently, especially in terms of brand preference (Ross *et al*., 2015), this review did not identify an influence upon measurable alcohol consumption.

Only 12 studies were longitudinal in design (seven of which were categorized as methodologically ‘strong’) and thus better able to examine the impact of alcohol marketing in early adolescence on subsequent behaviour (Smith and Foxcroft, 2009). Whilst most studies adjusted results for known predictors of drinking, it is impossible to determine whether all relevant confounding factors were accounted for. It is also not possible to rule out reciprocacy, i.e. whether alcohol consumption influences marketing rather than *vice versa*. Furthermore, this review may be subject to publication and reporting bias. It is impossible to predict the impact of unpublished data on our findings, and included papers may not have always reported null results or negative associations, especially where several research questions were addressed. Non-English language studies were excluded for practical reasons, and this may have excluded relevant literature from European countries in particular. Whilst most of the studies in this review took place in the USA, a small number were conducted in non high-income countries, which may reflect the increasing engagement of industry with developing alcohol markets (Jernigan and Babor, 2015). Several exposure measures included in these papers were not compatible with marketing regulations in high-income countries, including the provision of free alcohol by industry representatives to 11–16-year old adolescents (Swahn *et al*., 2011, 2013).

Additional exclusion criteria may have affected our findings. We excluded studies that examined general media portrayal as well as those focusing on product placement. The exposure of interest in this review was industry-led alcohol marketing. Whilst product placement is considered to be predominantly industry-led, it is difficult to disentangle this practice from general portrayal of alcohol products, resulting in little available data on drinking impact. Studies where assessments took place in an artificial environment were also excluded as they tended to include older adolescents and young adults and so were beyond the scope of this review. A recent systematic review included experimental studies, and assessed immediate effects of exposure to alcohol marketing on alcoholic beverage consumption and related cognitions among adults (predominantly undergraduate students). This work concluded that viewing alcohol advertisements (but not alcohol portrayals) may increase immediate alcohol consumption by small amounts (Stautz *et al*., 2016). Finally, studies were excluded from our review if the primary focus was exposure to brand-specific marketing as a predictor of market share rather than the impact of branding on young people's overall alcohol consumption (Siegel *et al*., 2011; Roberts *et al*., 2014; Ross *et al*., 2014a, b, 2015). We also excluded studies where purchasing behaviour was reported without measuring effects on consumption. As there are large discrepancies between survey-based measures of consumption and those based on alcohol sales (Bellis *et al*., 2009; Livingston and Callinan, 2015), it is not appropriate to infer consumption from purchasing behaviour.

## CONCLUSION

Previous systematic reviews have suggested that exposure to media and alcohol advertisements is associated with the likelihood that adolescents will start to drink alcohol, and with increased drinking among baseline drinkers. Despite the limitations outlined above, this systematic review also found a majority of evidence that linked industry-driven alcohol marketing (Price, Promotion, Product and Place) to key drinking outcomes (initiation, continuation, frequency and intensity) in young adolescents (aged 9–17 years). Nevertheless, we also found null results or negative associations. A field of highly variable and inconsistent exposure and outcome measures hampered our ability to conduct any data pooling. We did find a cluster of seven studies that focused on alcohol promotions exposure and ‘binge’ drinking outcomes. Yet these findings could not be pooled due to widely varying exposure measures (e.g. ownership of alcohol branded material, having a favourite advertisement, self-reported exposure to TV alcohol advertising). Future longitudinal research with standardized measures is needed to build on our work and enable robust effect size estimation in this field. Nevertheless, the volume and balance of evidence in this review provides sufficient confidence of an overall effect of promotional marketing (usually advertising) upon some early life drinking behaviours. Thus, taking a precautionary approach, we support recommendations of the WHO in its Global Alcohol Strategy that children and young people should be protected by strengthening advertising regulations (by limiting content to factual information and restricting scope to adult forums only) (World Health Organisation, 2010), as well as guidance from the UK's NICE recommending independent, ongoing monitoring of promotional practices by alcohol producers (National Institute for Clinical and Health Excellence, 2010).

## SUPPLEMENTARY MATERIAL

Supplementary material is available at* Alcohol and Alcoholism* online.

## FUNDING

This study was supported by the Economic and Social Research Council (ESRC) as part of a doctoral studentship (Research Centre Grant, UKCRC, Res 590-25-0004). S.S., E.K., C.M. and J.S. are members of Fuse—the Centre for Translational Research in Public Health, a UK Clinical Research Collaboration (UKCRC) Public Health Research Centre of Excellence. S.S., E.K. and J.S. are also supported by the National Institute for Health Research (NIHR)’s School for Public Health Research (SPHR). E.K. is also a member of the NIHR School of Primary Care Research. The views expressed in this paper are those of the authors and not necessarily those of the funders, UKCRC, the NHS, the NSPCC, the NIHR or the Department of Health.

## CONFLICT OF INTEREST STATEMENT

None declared.

## Supplementary Material

Supplementary Data

## References

[agw085C1] Alcohol Concern (2012) Making an Impression. Recognition of Alcohol Brands by Primary School Children. Cardiff: Alcohol Concern.

[agw085C2] Anderson P, Bruijn A, Angus K, et al (2009) Impact of alcohol advertising and media exposure on adolescent alcohol use: a systematic review of longitudinal studies. Alcohol Alcohol 44:229–43. 19144976 10.1093/alcalc/agn115

[agw085C110] Anderson P, Braddick F, Reynolds J, et al (eds.) (2012) Alcohol Policy in Europe: Evidence from AMPHORA. The AMPHORA project, available online: http://amphoraproject.net/view.php?id_cont=45

[agw085C3] Atkinson AM, Bellis M, Sumnall H (2013) Young peoples’ perspective on the portrayal of alcohol and drinking on television: findings of a focus group study. Addict Res Theory 21:91–9.

[agw085C4] Austin EW, Chin MJ, Grube JW (2006) How does alcohol advertising influence underage drinking? The role of desirability, identification and skepticism. J Adolesc Health 38:376–84. 16549298 10.1016/j.jadohealth.2005.08.017

[agw085C5] Austin EW, Knaus C (2000) Predicting the potential for risky behaviour among those ‘too young’ to drink as the result of appealing advertising. J Health Commun 5:13–27. 10848029 10.1080/108107300126722

[agw085C6] Azar D, White V, Coomber K, et al (2016) The association between alcohol outlet density and alcohol use among urban and regional Australian adolescents. Addiction 111:65–72. 26332165 10.1111/add.13143

[agw085C7] Bagnardi V, Rota M, Botteri E, et al (2015) Alcohol consumption and site-specific cancer risk: a comprehensive dose-response meta-analysis. Br J Cancer 112:580–93. 25422909 10.1038/bjc.2014.579 PMC4453639

[agw085C8] Baillie R (1996) Determining the effects of media portrayals of alcohol going beyond short-term influence. Alcohol Alcohol 31:235–42. 8844028 10.1093/oxfordjournals.alcalc.a008142

[agw085C9] Barry AE, Bates AM, Olusanya O, et al (2016) Alcohol marketing on Twitter and Instagram: evidence of directly advertising to youth/adolescents. Alcohol Alcohol 51:487–92. 26597794 10.1093/alcalc/agv128 PMC10150590

[agw085C10] Bellis MA, Hughes K, Cook PA, et al (2009) Off Measure: How We Underestimate the Amount We Drink. Liverpool: Centre for Public Health, Liverpool John Moores University.

[agw085C11] Bendtsen P, Damsgaard MT, Tolstrup JS, et al (2013) Adolescent alcohol use reflects community-level alcohol consumption irrespective of parental drinking. J Adolesc Health 53:368–73. 23763965 10.1016/j.jadohealth.2013.04.021

[agw085C12] Bonomo YA, Bowes G, Coffey C, et al (2004) Teenage drinking and the onset of alcohol dependence: a cohort study over seven years. Addiction 99:1520–8. 15585043 10.1111/j.1360-0443.2004.00846.x

[agw085C13] Bourdieu P (1990) The Logic of Practice. Cambridge: Polity Press.

[agw085C14] Brierley-Jones LK, Ling J, Wilson GB, et al (2014) Patterns of middle class alcohol use: habituses of ‘home’ and ‘traditional’ drinking. Social Health Illn 36:1054–76. 10.1111/1467-9566.12145 25060523

[agw085C15] Brown K (2016) Association between alcohol sports sponsorship and consumption: a systematic review. Alcohol Alcohol 51:747–55. 26911984 10.1093/alcalc/agw006 PMC5091292

[agw085C16] Browne-Yung K, Ziersch A, Baum F (2013) ‘Faking til you make it’: social capital accumulation of individuals on low incomes living in contrasting socio-economic neighbourhoods and its implications for health and wellbeing. Soc Sci Med 85:9–17. 23540360 10.1016/j.socscimed.2013.02.026

[agw085C17] Bryden A, Roberts B, McKee M, et al (2012) A systematic review of the influence on alcohol use of community level availability and marketing of alcohol. Health Place 18:349–57. 22154843 10.1016/j.healthplace.2011.11.003

[agw085C18] Chen MJ, Grube JW, Gruenewald PJ (2010) Community alcohol outlet density and underage drinking. Addiction 105:270–8. 20078485 10.1111/j.1360-0443.2009.02772.x PMC2810108

[agw085C19] Christensen VT, Carpiano RM (2014) Social class differences in BMI among Danish women: applying Cockerham's health lifestyles approach and Bourdieu's theory of lifestyle. Soc Sci Med 112:12–21. 24788112 10.1016/j.socscimed.2014.04.017

[agw085C20] Cockerham WC (2005) Health lifestyle theory and the convergence of agency and structure. J Health Soc Behav 46:51–67. 15869120 10.1177/002214650504600105

[agw085C21] Collins RL, Ellickson PL, McCaffrey D, et al (2007) Early adolescent exposure to alcohol advertising and its relationship to underage drinking. J Adolesc Health 40:527–34. 17531759 10.1016/j.jadohealth.2007.01.002 PMC2845532

[agw085C23] de Bruijn A, Engels R, Anderson P, et al (2016a) Exposure to online alcohol marketing and adolescents’ drinking: a cross-sectional study in four European countries. Alcohol Alcohol 51:615–21. 27151968 10.1093/alcalc/agw020

[agw085C24] de Bruijn A, Tanghe J, Beccaria F et al (2012) Report on the impact of European alcohol marketing exposure on youth alcohol expectancies and youth drinking Alcohol Measures for Public Health Research Alliance (AMPHORA).

[agw085C25] de Bruijn A, Tanghe J, de Leeuw J, et al (2016b) European longitudinal study on the relationship between adolescents’ alcohol marketing exposure and alcohol use. Addiction 111:1774–83. 27486952 10.1111/add.13455

[agw085C26] Demant J, Jarvinen M (2011) Social capital as norms and resources: focus groups discussing alcohol. Addict Res Theory 19:91–101.

[agw085C27] Dumsha J (2008) Changes in self-reported drinking behaviours among U.S. teenagers associated with the introduction of flavored malt beverages: an interrupted time series quasi-experiment. *Ph.D. Thesis*. TUI University.

[agw085C28] Dumsha J (2011) Changes in self-reported drinking behaviors among US teenagers associated with the introduction of flavored malt beverages: an interrupted time series quasi-experiment. Addict Res Theory 19:199–212.

[agw085C29] EACA (2009) EU advertising spend statistics.

[agw085C30] Ellickson P, Collins R, Hambarsoomians K, et al (2005) Does alcohol advertising promote adolescent drinking? Results from a longitudinal assessment. Addiction 100:235–46. 15679753 10.1111/j.1360-0443.2005.00974.x

[agw085C31] Faria R, Vendrame A, Silva R, et al (2011) Association between alcohol advertising and beer drinking among adolescents. Rev Saude Publica 45:441–7. 21484012 10.1590/s0034-89102011005000017

[agw085C32] Fisher LB, Miles IW, Austin SB, et al (2007) Predictors of initiation of alcohol use among US adolescents: findings from a prospective cohort study. Arch Pediatr Adolesc Med 161:959–66. 17909139 10.1001/archpedi.161.10.959

[agw085C33] Gordon R (2011) Critical social marketing: assessing the cumulative impact of alcohol marketing on youth drinking. *Ph.D. Thesis*. University of Stirling.

[agw085C34] Gordon R, Harris F, Mackintosh AM, et al (2010a) Assessing the cumulative impact of alcohol marketing on young people's drinking: cross-sectional data findings. Addict Res Theory 19:66–75.

[agw085C35] Gordon R, MacKintosh AM, Moodie C (2010b) The impact of alcohol marketing on youth drinking behaviour: a two-stage cohort study. Alcohol Alcohol 45:470–80. 20739441 10.1093/alcalc/agq047

[agw085C36] Gore FM, Bloem PJN, Patton GC, et al (2011) Global burden of disease in young people aged 10–24 years: a systematic analysis. Lancet 377:2093–102. 21652063 10.1016/S0140-6736(11)60512-6

[agw085C37] Grant BF, Stinson FS, Harford TC (2001) Age at onset of alcohol use and DSM-IV alcohol abuse and dependence: a 12-year follow-up. J Subst Abuse 13:493–504. 11775078 10.1016/s0899-3289(01)00096-7

[agw085C38] Grenard JL (2008) Exposure to alcohol advertising on television and alcohol use among young adolescents (dissertation). University of Southern California.

[agw085C39] Grenard JL, Dent CW, Stacy AW (2013) Exposure to alcohol advertisements and teenage alcohol-related problems. Pediatr 131:e369–79. 10.1542/peds.2012-1480 PMC3557408 23359585

[agw085C40] Gupta H, Pettigrew S, Lam T, et al (2016) A systematic review of the impact of exposure to internet-based alcohol-related content on young people's alcohol use behaviours. Alcohol Alcohol 51:763–71. 27522028 10.1093/alcalc/agw050

[agw085C41] Hastings G, Anderson S, Cooke E, et al (2005) Alcohol marketing and young people's drinking: a review of the research. J Public Health Policy 26:296–311. 16167558 10.1057/palgrave.jphp.3200039

[agw085C42] Hastings G, Brooks O, Stead M, et al (2010) Alcohol advertising: the last chance saloon. BMJ 340:b5650. 20089576 10.1136/bmj.b5650

[agw085C43] Henriksen L, Feighery EC, Schleicher NC, et al (2008) Receptivity to alcohol marketing predicts initiation of alcohol use. J Adolesc Health 42:28–35. 18155027 10.1016/j.jadohealth.2007.07.005 PMC2175037

[agw085C44] Holmes J, Guo Y, Maheswaran R, et al (2014a) The impact of spatial and temporal availability of alcohol on its consumption and related harms: a critical review in the context of UK licensing policies. Drug Alcohol Rev 33:515–25. 25186193 10.1111/dar.12191 PMC4313683

[agw085C45] Holmes MV, Dale CE, Zuccolo L, et al (2014b) Association between alcohol and cardiovascular disease: Mendelian randomisation analysis based on individual participant data. BMJ 349:1–16. 10.1136/bmj.g4164 PMC4091648 25011450

[agw085C46] Huckle T, Huakau J, Sweetsur P, et al (2008) Density of alcohol outlets and teenage drinking: living in an alcogenic environment is associated with higher consumption in a metropolitan setting. Addiction 103:1614–21. 18821871 10.1111/j.1360-0443.2008.02318.x

[agw085C47] Jarvinen M, Gundelach P (2007) Teenage drinking, symbolic capital and distinction. J Youth Stud 10:55–71.

[agw085C48] Jernigan D, Noel J, Landon J, et al (2016) Alcohol marketing and youth alcohol consumption: a systematic review of longitudinal studies published since 2008. Addiction, doi:10.1111/add.13591. 10.1111/add.13591 27565582

[agw085C49] Jernigan DH, Babor TF (2015) The concentration of the global alcohol industry and its penetration in the African region. Addiction 10:551–60. 10.1111/add.12468 25771689

[agw085C50] Jones SC, Magee CA (2011) Exposure to alcohol advertising and alcohol consumption among australian adolescents. Alcohol Alcohol 46:630–7. 21733835 10.1093/alcalc/agr080

[agw085C51] Kuntsche E, Kuendig H, Gmel G (2008) Alcohol outlet density, perceived availability and adolescent alcohol use: a multilevel structural equation model. J Epidemiol Community Health 62:811–6. 18701732 10.1136/jech.2007.065367

[agw085C52] Kuntsche E, Rossow I, Simons-Morton B, et al (2013) Not early drinking but early drunkenness is a risk factor for problem behaviours among adolescents from 38 European and North American countries. Alcohol Clin Exp Res 37:308–314. 23240610 10.1111/j.1530-0277.2012.01895.x PMC4169008

[agw085C53] Lin EY, Caswell S, You RQ, et al (2012) Engagement with alcohol marketing and early brand allegiance in relation to early years of drinking. Addict Res Theory 20:329–38.

[agw085C54] Livingston M, Callinan S (2015) Underreporting in alcohol surveys: whose drinking is underestimated. J Stud Alcohol Drugs 76:158–64. 25486405

[agw085C55] Lo CC, Weber J, Cheng TC (2013a) A spatial analysis of student binge drinking, alcohol-outlet density and social disadvantages. Am J Addict 22:391–401. 23795880 10.1111/j.1521-0391.2013.12022.x PMC3694625

[agw085C56] Lo CC, Weber J, Cheng TC (2013b) Urban-rural differentials: a spatial analysis of Alabama students’ recent alcohol use and marijuana use. Am J Addict 22:188–96. 23617858 10.1111/j.1521-0391.2012.12023.x PMC3641678

[agw085C57] Lunnay B, Ward P, Borlagdan J (2011) The practise and practice of Bourdieu: the application of social theory to youth alcohol research. Int J Drug Policy 22:428–36. 21862305 10.1016/j.drugpo.2011.07.013

[agw085C58] Maimaris W, McCambridge J (2013) Age of first drinking and adult alcohol problems: systematic review of prospective cohort studies. J Epi Comm Health 68:268–74. 10.1136/jech-2013-203402 PMC4158030 24249000

[agw085C59] McClure AC, Dal Cin S, Gibson J, et al (2006) Ownership of alcohol-branded merchandise and initiation of teen drinking. Am J Prev Med 30:277–83. 16530613 10.1016/j.amepre.2005.11.004

[agw085C60] McClure AC, Stoolmiller M, Tanski SE, et al (2013) Alcohol marketing receptivity, marketing-specific cognitions and underage binge drinking. Alcohol Clin Exp Res 37:E404–13. 23256927 10.1111/j.1530-0277.2012.01932.x PMC3548023

[agw085C61] McClure AC, Stoolmiller M, Tanski SE, et al (2009) Alcohol-branded merchandise and its association with drinking attitudes and outcomes in US adolescents. Arch Pediat Adol Med 163:211–7. 10.1001/archpediatrics.2008.554 PMC2707771 19255387

[agw085C62] McCreanor T, Barnes H, Kaiwai H, et al (2008) Creating intoxigenic environments: marketing alcohol to young people in Aotearoa New Zealand. Soc Sci Med 67:938–46. 18619720 10.1016/j.socscimed.2008.05.027

[agw085C63] Meier P, Booth A, Brennan A et al (2008) *The independent review of the effects of alcohol pricing and promotion*. School of Health and Related Research, University of Sheffield.

[agw085C64] Meier PS, Purshouse R, Brennan A (2009) Policy options for alcohol price regulation: the importance of modelling population heterogeneity. Addiction 105:383–93. 19839965 10.1111/j.1360-0443.2009.02721.x

[agw085C65] Moher D, Liberati A, Tetzlaff J, et al. and PRISMA group (2009) Preferred reporting items for systematic reviews and meta-analyses: the PRISMA statement. Ann Intern Med 151:264–9. 19622511 10.7326/0003-4819-151-4-200908180-00135

[agw085C66] Moreno MA, Christakis DA, Egan KG, et al (2012) Associations between displayed alcohol references on Facebook and problem drinking among college students. Arch Pediat Adol Med 166:157–63. 10.1001/archpediatrics.2011.180 PMC3266463 21969360

[agw085C67] Morgenstern M, Isensee B, Sargent JD, et al (2011) Attitudes as mediators of the longitudinal association between alcohol advertising and youth drinking. Arch Pediat Adol Med 165:610–6. 10.1001/archpediatrics.2011.12 21383258

[agw085C68] Morgenstern M, Sargent JD, Sweeting H, et al (2014) Favourite alcohol advertisements and binge drinking among adolescents: a cross-cultural cohort study. Addiction 109:2005–15. 24962215 10.1111/add.12667

[agw085C69] National Collaborating Centre for Methods and Tools (1998) Quality Assessment Tool for Quantitative Studies. Hamilton, ON: McMaster University 2008 [updated April 2010; cited 2014 April]. Available from: http://www.nccmt.ca/registry/view/eng/15.html.

[agw085C70] National Institute for Clinical and Health Excellence (2010) Alcohol use disorders: preventing the development of hazardous and harmful drinking. NICE public health guidance 24. London: National Institute for Health and Clinical Excellence.

[agw085C71] Nicholls J (2012) Everyday, everywhere: alcohol marketing and social media—current trends. Alcohol Alcohol 47:486–93. 22532575 10.1093/alcalc/ags043

[agw085C72] Noel JK, Babor TF, Robaina K (2016) Industry self-regulation of alcohol marketing: a systematic review of content and exposure research. Addiction, doi:10.1111/add.13410. 10.1111/add.13410 27188217

[agw085C73] Pasch KE, Hearst MO, Nelson MC, et al (2009) Alcohol outlets and youth alcohol use: exposure in suburban areas. Health Place 15:642–6. 19084464 10.1016/j.healthplace.2008.10.002 PMC2739405

[agw085C74] Paschall MJ, Grube JW, Black C, et al (2007) Is commercial alcohol availability related to adolescent alcohol sources and alcohol use? Findings from a multi-level study. J Adolesc Health 41:168–74. 17659221 10.1016/j.jadohealth.2007.03.009 PMC2213632

[agw085C75] Reboussin BA, Song EY, Wolfson M (2011) The impact of alcohol outlet density on the geographic clustering of underage drinking behaviours within census tracts. Alcohol Clin Exp Res 35:1541–9. 21463343 10.1111/j.1530-0277.2011.01491.x PMC3132245

[agw085C76] Ridout B, Campbell A, Ellis L (2012) ‘Off your Face(book)’: alcohol in online social identity construction and its relation to problem drinking in university students. Drug Alcohol Rev 31:20–6. 21355935 10.1111/j.1465-3362.2010.00277.x

[agw085C77] Roberts SP, Siegel MB, DeJong W, et al (2014) The relationships between alcohol source, autonomy in brand selection and brand preference among youth in the USA. Alcohol Alcohol 49:563–71. 25113176 10.1093/alcalc/agu034 PMC4128668

[agw085C78] Ross C, Ostroff J, Siegel MB, et al (2014a) Youth alcohol brand consumption and exposure to brand advertising in magazines. J Stud Alcohol Drugs 75:615–22. 24988260 10.15288/jsad.2014.75.615 PMC4108602

[agw085C79] Ross CS, Maple E, Siegel M, et al (2014b) The relationship between brand-specific alcohol advertising on television and brand-specific consumption among underage youth. Alcohol Clin Exp Res 38:2234–42. 24986257 10.1111/acer.12488 PMC4146644

[agw085C80] Ross CS, Maple E, Siegel M, et al (2015) The relationship between population-level exposure to alcohol advertising on television and brand-specific consumption among underage youth in the US. Alcohol Alcohol 50:358–64. 25754127 10.1093/alcalc/agv016 PMC4398991

[agw085C81] Rowland B, Toumbourou JW, Satyen L, et al(2014) Associations between alcohol outlet densities and adolescent alcohol consumption: a study in Australian students. Addict Behav 39:282–8. 24183302 10.1016/j.addbeh.2013.10.001

[agw085C82] Saffer H, Dave D (2006) Alcohol advertising and alcohol consumption by adolescents. Health Econ 15:617–37. 16475245 10.1002/hec.1091

[agw085C83] Scott S, Baker R, Shucksmith J, et al (2014) Autonomy, special offers and routines: a Q methodological study of industry-driven marketing influences on young people's drinking behaviour. Addiction 109:1833–44. 24938633 10.1111/add.12663

[agw085C84] Seaman P, Edgar F, Ikegwuonu T (2013) The role of alcohol price in young adult drinking cultures in Scotland. Drugs 20:278–85. 23864771 10.3109/09687637.2013.765386 PMC3709883

[agw085C85] Secretan B, Straif K, Baan R, et al (2009) A review of human carcinogens—Part E: tobacco, areca nut, alcohol, coal smoke, and salted fish. Lancet Oncol 10:1033–4. 19891056 10.1016/s1470-2045(09)70326-2

[agw085C86] Shamblen SR, Harris MS, Ringwalt CL, et al (2011) Outlet density as a predictor of alcohol use in early adolescence. Subst Use Misuse 46:1049–59. 21345047 10.3109/10826084.2011.552933

[agw085C87] Sheron N, Gilmore I (2016) Effect of policy, economics, and the changing alcohol marketplace on alcohol related deaths in England and Wales. BMJ 353:i1860. 27052417 10.1136/bmj.i1860

[agw085C88] Siegal M, DeJong W, Naimi TS, et al (2013) Brand-specific consumption of alcohol among underage youth in the United States. Alcohol Clin Exp Res 37:1195–203. 23398328 10.1111/acer.12084 PMC3655157

[agw085C89] Siegel M, DiLoreto J, Fortunato EK, et al (2011) Development and pilot testing of an internet-based survey instrument to measure the alcohol brand preferences of U.S. youth. Alcohol Clin Exp Res 35:765–72. 21223311 10.1111/j.1530-0277.2010.01394.x

[agw085C90] Smith LA, Foxcroft D (2009) The effect of alcohol advertising, marketing and portrayal on drinking behaviour in young people: systematic review of prospective cohort studies. BMC Public Health 9:1–11. 19200352 10.1186/1471-2458-9-51 PMC2653035

[agw085C91] Stacy AW, Zogg JB, Unger JB, et al (2004) Exposure to televised alcohol ads and subsequent adolescent alcohol use. Am J Health Behav 28:498–509. 15569584 10.5993/ajhb.28.6.3

[agw085C92] Stanley LR, Henry KL, Swaim RC (2011) Physical, social and perceived availabilities of alcohol and last month alcohol use in rural and small urban communities. J Youth Adolesc 40:1203–14. 20532969 10.1007/s10964-010-9556-z PMC9128659

[agw085C93] Stautz K, Brown KG, King SE, et al (2016) Immediate effects of alcohol marketing communications and media portrayals on consumption and cognition: a systematic review and meta-analysis of experimental studies. BMC Public Health 16:1–18. 27278656 10.1186/s12889-016-3116-8 PMC4899920

[agw085C94] Stoolmiller M, Wills TA, McClure AC, et al (2012) Comparing media and family predictors of alcohol use: a cohort study of US adolescents. BMJ Open 2:e000543. 10.1136/bmjopen-2011-000543 PMC3289988 22349939

[agw085C95] Swahn MH, Ali B, Palmier JB, et al (2011) Alcohol marketing, drunkenness, and problem drinking among Zambian youth: findings from the 2004 Global School-based Student Health Survey. J Environ Public Health 2011:497827. 21647354 10.1155/2011/497827 PMC3103909

[agw085C96] Swahn MH, Palmier JB, Benegas-Segarra A, et al (2013) Alcohol marketing and drunkenness among students in the Philippines: findings from the nationally representative Global School-based Student Health Survey. BMC Public Health 13:1159. 24325264 10.1186/1471-2458-13-1159 PMC3890547

[agw085C97] Tobler AL (2009) Neighbourhood context and alcohol use among urban, low-income, multi-ethnic, young adolescents. *Ph.D. Thesis*. University of Florida.

[agw085C98] Tobler AL, Komro KA, Maldonado-Mollina MM (2009) Relationship between neighbourhood context, family management practices and alcohol use among urban, multi-ethnic, young adolescents. Prev Sci 10:313–24. 19381808 10.1007/s11121-009-0133-1 PMC2783307

[agw085C99] Tobler AL, Livingston MD, Komro KA (2011) Racial/ethnic differences in the etiology of alcohol use among urban adolescents. J Stud Alcohol Drugs 72:799–810. 21906507 10.15288/jsad.2011.72.799 PMC3174025

[agw085C100] Townshend T (2013) Youth, alcohol and place-based leisure behaviours: a study of two locations in England. Soc Sci Med 91:153–61. 23477845 10.1016/j.socscimed.2013.02.017

[agw085C101] Truong KD (2008) Essays on environmental determinants of health behaviours and outcomes. *Ph.D. Thesis*. Pardee RAND Graduate School.

[agw085C102] Truong KD, Sturm R (2009) Alcohol environments and disparities in exposure associated with adolescent drinking in California. Am J Public Health 99:264–70. 19059870 10.2105/AJPH.2007.122077 PMC2622793

[agw085C103] van Hoof JJ, van Noordenburg M, de Jong M (2008) Happy hours and other alcohol discounts in cafés: prevalence and effects on underage adolescents. J Public Health Policy 29:340–52. 18701902 10.1057/jphp.2008.2

[agw085C104] Williams R, Aspinall R, Bellis M, et al (2014) Addressing liver disease in the UK: a blueprint for attaining excellence in health care and reducing premature mortality from lifestyle issues of excess consumption of alcohol, obesity and viral hepatitis. Lancet 384:1953–97. 25433429 10.1016/S0140-6736(14)61838-9

[agw085C105] Witt E (2010) Research on alcohol and adolescent brain development: opportunities and future directions. Alcohol Alcohol 44:119–24. 10.1016/j.alcohol.2009.08.011 20113880

[agw085C106] Workman JE (2003) Alcohol promotional clothing items and alcohol use by underage consumers. Fam Consum Sci Res J 31:331–54.

[agw085C107] World Health Organisation (2010) Global strategy to reduce the harmful use of alcohol. http://www.who.int/substance_abuse/activities/gsrhua/en/ (20 October 2016, date last accessed).

[agw085C108] Young R, Macdonald L, Ellaway A (2013) Associations between proximity and density of local alcohol outlets and alcohol use among Scottish adolescents. Health Place 19:124–30. 23220375 10.1016/j.healthplace.2012.10.004 PMC3885793

[agw085C109] Zogg J (2004) Adolescent exposure to alcohol advertising: a prospective extension of Strickland's model (dissertation). University of Southern California.

