# Supplementary material for: Does Industry-Driven Alcohol Marketing Influence Adolescent Drinking Behaviour? A Systematic Review
Source: Alcohol Alcohol. 2016 Dec 20;52(1):84–94. doi: 10.1093/alcalc/agw085 (PMC5169036; doi:10.1093/alcalc/agw085)
Supplement: Supplementary Data [file supp_agw085_agw085.DC1.html]

Supplementary Data | Alcohol and Alcoholism

## Supplementary Data

Supplementary Data

- Supplementary Data - docx file
