# Supplementary material for: Does Industry-Driven Alcohol Marketing Influence Adolescent Drinking Behaviour? A Systematic Review
Source: Alcohol Alcohol. 2016 Dec 20;52(1):84–94. doi: 10.1093/alcalc/agw085 (PMC5169036; doi:10.1093/alcalc/agw085)
Supplement: Supplementary Data [file SupplementaryFilesScottetal.docx]

**Supplementary File**

**Abbreviations**

ABI Alcohol Branded Item

ABM Alcohol Branded Merchandise

AHR Adjusted Hazard Ratio

AOR Adjusted Odds Ratio

APCI Alcohol Promotional Clothing Item

API Alcohol Promotional Item

CI Confidence Interval

HR Hazard Ratio

MTF Monitoring the Future

NLSY National Longitudinal Survey of Youth

OR Odds Ratio

QF Quantity Frequency (drinking volume)

RSOD Risky Single Occasion Drinking

SD Standard Deviation

SE Standard Error

SEIFA Socio Econmic Indexes for Areas

TP Time Point

**Table S1: Summary of included studies**

| **Study and setting** | **Age (years)**  **Sample** | **Gender** | **SES** | **Ethnic background** | **Study design;**  **Quality rating** | **Exposure Measure(s)** | **Behavioural Outcome Measure(s)** | **Follow up (rate / duration)** | **Reported analyses** |
| --- | --- | --- | --- | --- | --- | --- | --- | --- | --- |
| Azar *et al* (2016); Australia; Community | 12-17  (n=68,208) | %male metropolitan areas:  TP1: 49.1  TP2: 49.9  TP3: 49.5  TP4: 49.4  %male rural areas:  TP1: 49.4  TP2: 50.9  TP3: 52.3  TP4: 50.5 | %SEIFA quintile 1 metropolitan areas:  TP1: 13.2  TP2: 14.3  TP3: 17.0  TP4: 12.3  %SEIFA quintile 1 rural areas:  TP1: 21.6  TP2: 20.1  TP3: 35.0  TP4: 28.6 | %Indigenous metropolitan areas:  TP1: 3.1  TP2: 3.1  TP3: 3.2  TP4: 3.1  %Indigenous rural areas:  TP1: 6.5  TP2: 5.2  TP3: 5.6  TP4: 7.8 | Cross-sectional; collected at four TPs **(Weak)** | Postcode-level outlet density (no. of licenses per 1000 population) for each outlet type (general, on-premise, off-premise, clubs). | Past month alcohol use (yes/no); risky drinking in past 7 days (≥5 drinks on at least 1 occasion) amongst all students and past week drinkers | Four waves collected at 3 year intervals | Mixed-effects logistic regression |
| Bendtsen *et al* (2013); Denmark; School | Mean: 13.7; 15.7 (n=2911) | M=1,454  F=1,457 | Family structure:  Living with both biological parents: n=1,820  Other family type: n=921 | Danes: n=2,548  Descendents / immigrants: n=363 | Cross-sectional **(Weak)** | Self-reported no. of alcohol outlets near school (≥2/ 1 /0) | Lifetime drunkenness (assessed with ‘have you ever been really drunk’; categorized as no / never / yes, once / yes, 2-3 times / yes, 4-10 times / yes, ≥10 times; dichotomised to twice or more/seldom) | None | Multilevel regression |
| Chen *et al* (2010);  USA; Community | 14-16 at baseline (mean=14.9) (n=1091) | %male: 53 | Zip codes stratified into high/medium/low based on household income data from 2000 Census (data not reported) | Latino: 33%  Non-Hispanic white: 51%  Other: 16% | Longitudinal **(Weak)** | Off-premise outlet counts per 1000 roadway miles at zip code level | Drinking frequency in past 12 months (no. of days consumed at least a whole drink of alcohol); frequency of excessive drinking (no. of days drunk) | Three waves collected at 1 year intervals | Three-level linear growth model |
| Collins *et al* (2007)  USA; School | Mean: 11.8 years at baseline  (n=1786) | %female: 51 | Parental Education:  Mean: 3.33  SD: 1.04 | White: 85%  Native American: 12%  Other: 3% | Cross-sectional; collected at two TPs **(Weak)** | 1. Exposure to beer adverts on ESPN (weighted measure based on 5-point frequency of watching televised pro football, pro basketball, college football, college basketball, and NASCAR races; days per week watched ESPN; and past month viewing of ESPN sports center)  2. Exposure to beer adverts on other sports programmes (weighted responses to the 5-sports item using Nielsen data, but excluding adverts aired on ESPN or ESPN2)  3. Exposure to beer adverts on non-sports TV programmes (frequency of watching each weighted by the relevant no. of adverts, items summed, and total divided by 1000)  4. Exposure to alcohol advertising in magazines (past year frequency of looking at Rolling Stone, Sports Illustrated, People, Field and Stream, and Newsweek, on five-point scales)  5.Past year exposure to beer concession stands (7-point scale)  6. Past year exposure to in-store beer displays (7-point scale)  7. Ownership of APIs (yes/no) | Past year beer drinking (any/none) | 12 months | Multivariate regression |
| de Bruijn *et al* (2012; 2013; 2016a; 2016b); Germany; Italy; Netherlands; Poland; School | TP1: mean: 14.05yrs  (n=9038)  TP1 and TP2: mean: 13.95yrs (n=6651)  TP3: mean: 14yrs (n=9075) | 2013 and 2016a (TP1 only): 50% male  2012 (TP1 and TP2): 51.1% female  2016b (TP3): 49.5% male | 2016b (TP3):  Education (%):  General level: 28  Lowest level: 26  Intermediate level: 22  Highest level: 24 | Not reported | Longitudinal; collected at three TPs **(Strong)** | 1. Frequency of exposure to alcohol marketing in online media (never / rarely / sometimes / often / very often; categorized 1-5)  2. Ownership of ABIs (yes/no)  3. Frequency of exposure to TV alcohol advertising, measured as frequency of viewing 8 selected TV programmes (total score of between 0 and 1 calculated for each respondent)  4. 13-item latent variable measuring exposure to online alcohol marketing, televised alcohol marketing, alcohol sport sponsorship, music event/festival sponsorship, ownership of ABIs, reception of free samples and exposure to price offers. | TP1: Onset of binge drinking (≥5 drinks in a single occasion in last 30 days; yes/no)  TP1, TP2 and TP3: Frequency of binge drinking (≥5 drinks in a single occasion) in last 30 days (0; 1; 2; 3-5; 6-9; ≥10)  TP1, TP2 and TP3: Frequency of alcohol consumption in last 30 days (0; 1-2; 3-5; 6-9; 10-19; ≥20) | Three waves (14-17 month period between TP1 and TP3) | TP1: Binary and logistic regression  TP1 and TP2: hierarchical regression  TP1, TP2 and TP3: auto-regressive cross-lagged modelling |
| Dumsha (2008; 2011); USA; Country | ≤14: n=5547  15: n=13353  16: n=15532  17: n=15871 | F=30,519  M=29,702 | Not reported | White not hispanic: n=23882  Black not hispanic: n=15045  Hispanic or Latino: n=14675  Asian / Pacific Islander: n=6237 | Interrupted time-series  **(Moderate)** | Introduction of alcopops to the US beverage market in 1999 | Lifetime drinking (no. days consumed at least one drink of alcohol); age at first full drink; no. days consumed at least one drink of alcohol in past 30 days; no. days of binge drinking in past 30 days (≥5 drinks within a couple of hours). | None | Segmented (linear) regression |
| Ellickson *et al* (2005); USA; School | 12 at baseline; 13-15 at follow-up  (n=3,111) | F=50%  M=50% | Not reported | White:88%  N. American: 6.3%  Other: 5.4% | Prospective cohort **(Moderate)** | 1. Exposure to TV sport / late night programming that air beer adverts over a 7-month period (never / some of the time / half the time / most of the time / almost all the time)  2. Frequency of reading 6 pre-selected magazines that advertise alcohol over past year (5-point scale, ranging never to ≥10 for monthly magazines and never to ≥31 for weekly magazines)  3. Exposure to beer concession stands over past year (7-point scale ranging never to ≥3 times per week)  4. Exposure to in-store beer displays over past year ((7-point scale ranging never to ≥3 times per week) | Baseline drinking status (drinkers/non-drinkers); drinking in past 12 months at follow up (none / 1-2 times / 3-10 times / 11–20 times / ≥20 times) | Three waves | Logistic regression |
| Faria *et al* (2011);  Brazil; School | 11-16  (n=1115) | F=53.3% (n=594)  M=46.7% (n=520) | 92.6% (n=1007) had a bathroom at home. | Not reported | Cross-sectional  **(Weak)** | Favourite alcohol brand (yes/no); attention to alcohol advertisements (yes/no); belief that advertisements tell the truth (true/false); belief that parties attended are similar to those seen in commercials (true/false) | Beer drinking in the last 30 days (yes/no) | None | Univariate and multiple logistic regression |
| Fisher *et al* (2007);  USA; School | 11-18  (n=5511) | M=2228 (40%)  F=3283 (60%) | Not reported; mothers all hold nursing degrees. | Predominantly white (94%). | Prospective cohort **(Strong)** | Awareness of alcohol advertising (yes/no); owning / being willing to use APIs (yes/no) | Ever tried alcohol (yes/no); consumption of whole drink of alcohol (yes/no); binge drinking in past year (≥5 drinks within a few hours; yes/no). | 12 months | Logistic regression |
| Gordon *et al* (2010a; 2010b; 2011); UK; Home | 12-14 (mean=13) at baseline; 14-16 (mean=15) at follow up  (n=552) | M= 50% (n=275)  F=50% (n=277) | ABC1 (middle class) = 41% (n=224)  C2DE (working class) = 59% (n=326) | White = 94% (n=515)  Asian = 3% (n=19)  Mixed Race =1% (n=7)  Black = 1% (n=6)  Chinese = <1% (n=1)  Other = <4% (n=1) | Cross-sectional cohort data **(Strong)** | 1. Marketing awareness assessed for 15 types of marketing (yes/no/don’t know; count of positive responses)  2. Marketing involvement (free product samples; free gifts showing alcohol brand logos; special alcohol price offers; promotional mail or e-mails mentioning alcohol brands; owned clothing or other ABIs; looked at alcohol brand websites; downloaded alcohol-branded electronic content; used social networking sites containing alcohol brands or logos; yes/no/don’t know; count of positive responses)  3. Marketing appreciation (5-point scale: ‘dislike a lot’ to ‘like a lot’) | Drinking status (drinkers / non-drinkers); age at first drink; uptake of drinking (baseline non-drinkers who were drinkers at follow-up / baseline non-drinkers who remained non-drinkers at follow-up) units of alcohol last consumed; frequency of drinking (daily / twice per week / weekly / fortnightly / monthly / a few times per year / never) | 2 TPs; follow up data collected two years later | Logistic and multiple regressions |
| Grenard *et al* (2008; 2013); USA; School | 12-16  (n=3,890) surveyed in at least one wave | M=1894 (49.86%)  F=1905 (50.14%) | Parent Education:  Father:  Mean: 3.52  SD: 1.63  Mother:  Mean: 3.49  SD: 1.57 | Non-Hispanic Whites: 13.37%  Latino: 47.87% Asian: 17.02% African American: 3.08%  Native Hawaiian or Pacific Islander: 0.77%  American Native: 0.95%  Mixed: 4.32%  Didn’t know: 12.62% | Prospective cohort **(Weak)** | 1. Past month exposure to alcohol advertising during popular TV shows (6-point scale ranging ‘never’ to ‘every day’)  2. Past month exposure to alcohol advertising during sports programs (6-point scale ranging ‘never’ to ‘every day’)  3. Self-reported exposure to TV alcohol advertising (4 items: 1. how often seen alcohol adverts, 5-point scale ranging ‘a lot’ to ‘never’; 2. how many alcohol adverts seen in past week, 6-point scale ranging 0-6; 3. whether seen a beer advert in past 6 months, 7-point scale ranging ‘every day’ to ‘never’; 4. whether seen a wine/liquor advert in past 6 months, 7-point scale ranging ‘every day’ to ‘never’)  4. Liking of alcohol adverts (funny, 5-point scale ranging ‘yes always’ to ‘never see any’; sexy, 5-point scale ranging ‘yes always’ to ‘never see any’; and liking of alcohol adverts in comparison to all adverts (5-point scale ranging ‘like the most’ to ‘never seen a TV alcohol advert’) | Alcohol use in the past 30 days (5-items) / past 6 months (4-items) to form an index using all 9 items | 4 TPs | Structural equation models (4 models) |
| Henriksen *et al* (2008); USA; School | 10-15  (n=1080) | %female: 57.3 | Not reported | Non-Hispanic White: 28%  Hispanic / Latino: 39%  Other / unknown: 33% | Cross-sectional; collected at two TPs **(Weak)** | API ownership (yes/no); receptivity to owning/using an API (yes/no); brand in favourite alcohol advertisement (open question); used to calculate high, moderate and low level of alcohol marketing receptivity; brand recall (min 0, max 7) | 1. Drinking initiation assessed using 3 items: 1. ever finished all/most of an alcoholic drink; 2. No. of days in the past 30 ever finished all/most of an alcoholic drink; 3. No. of days in the past 7 ever finished all/most of an alcoholic drink (never/ever drinking – any alcohol use coded as drinking initiation)  2. Transition from never to current drinking (1-2 drinking days in the past month) | 12 months | Logistic regression |
| Huckle *et al* (2008)  New Zealand; Community | 12-17  (n=1179) | M= 52%  F = 48% | Not reported | European:63%  Maori:12%  Pacific people: 8%  Asian: 16%  Other:1% | Cross-sectional **(Moderate)** | Outlet density count per census area unit (median population: 2000) | Typical occasional quantity; frequency of drinking and frequency of drunkenness in past 12-months (count) | None | Multi-level modelling; logistic regression |
| Jones and Magee (2011); Australia; Community | 12-17  (n=1113) | M=446 (40.1%)  F=667 (59.9%) | Not reported | Not reported | Cross-sectional **(Weak)** | Exposure to alcohol advertising (ever) via: television (yes/no), newspapers (yes/no), magazines (yes/no), bars or pubs (yes/no), billboards/posters (yes/no), internet and promotional materials (yes/no), advertisements in bottleshops / liquor stores (yes/no) | Ever consumed alcohol (never/few sips/ <10 drinks/ >10 drinks); frequency in past 12 months (less than monthly/more than monthly); consumption in past 4 weeks (no consumption/some consumption) | None | Logistic regression |
| Kuntsche *et al* (2008); Switzerland; School | 12-17  (mean=14.8)  (n=6183) | M= 3070 (49.7%)  F = 3113 (50.3%) | Not reported | Not reported | Cross-sectional **(Moderate)** | Outlet density count of on and off premises per 1000 habitants | Past 12-month QF (no. of drinks consumed in a typical occasion multiplied by frequency of alcohol use; ranging <1 to ≥5 drinks); RSOD over past 30 days (≥5 drinks in one occasion, ranging from 0 to ≥10 times) | None | Two-level structural equation model; logistic regression |
| Lin *et al* (2012);  New Zealand; Telephone | 12-15  (n=2538) | M=1302 (51.3%)  F=1236 (48.7%) | Not reported | Not reported | Cross-sectional  **(Moderate)** | 1. Marketing awareness assessed for 15 types of marketing (yes/no/don’t know; count of positive responses)  2. Marketing engagement (free product samples; free gifts showing alcohol brand logos; special alcohol price offers; promotional mail or e-mails mentioning alcohol brands; owned clothing or other ABIs; looked at alcohol brand websites; downloaded alcohol-branded electronic content; used social networking sites containing alcohol brands or logos; yes/no/don’t know; count of positive responses)  3. Favourite alcohol brand (yes/no) | Drinking in past 12 months (yes/no); no. of drinking occasions per year; typical occasion volume consumption (units) | None | Logistic and linear regression |
| Lo *et al* (2013a; 2013b); USA; School | Mean=14.5  Study 1: n=78,138  Study 2: n=92,822 | Male:  Mean: 0.456  SD: 0.498 | Not reported | White: 64.2% African American: 28.8%  Other:7% | Cross-sectional **(Moderate)** | School catchment level alcohol outlet density count for three variables - bars, retail stores and restaurants (calculated per each square km) | Frequency of binge drinking in past 2 weeks (≥5 drinks in 2 hours; 7-point scale, 0 occasions, 1-2, 3-5, 6-9, 10-19, 20-39, ≥40); 30-day alcohol use (7-point scale, 0 occasions, 1-2, 3-5, 6-9, 10-19, 20-39, ≥40) | None | Multi-level modelling; hierarchical linear regression |
| McClure *et al* (2009; 2013); Stoolmiller *et al* (2012); USA; Telephone | 10-14 at baseline  n=6552 (baseline)  n=5503 (8 months)  n=5019 (16 months)  n=4575 (24 months)  n=1734 (6^th^ wave) | M=3350 (51%)  F=3172 (49%) | Parent education: Less than a high school degree (17%); High school degree only (23%); Post high school education but no degree (21%); associate degree (9%); bachelor’s degree (18%); education beyond a bachelor’s degree (12%). | White = 62%  Black = 11%  Hispanic = 19%  Mixed race / other = 8% | Longitudinal cohort; cross-sectional analysis at 6^th^ wave (2013) **(Moderate)** | Ownership of ABM at 8, 16 and 24 months (yes/no); favourite advertisement (yes/no; 6^th^ wave data only) | Initiation of alcohol use by follow-up that parents did not know about (yes/no); binge drinking (≥5 drinks in a row within a couple of hours; yes/no); 30-day binge drinking (yes/no) | 4 waves over 24 months; cross-sectional analysis of wave 6 data | Panel and hazard logistic regression |
| McClure *et al* (2006); USA; Telephone | 10-14 at baseline  (n=2406) | M=1111 (46%)  F=1295 (54%) | Parent education; Neither or one completed high school = 383 (16%); Both completed high school = 2023 (84%) | Primarily Caucasian (95%) | Longitudinal cohort **(Strong)** | Ownership of ABM at follow-up (yes/ no / don’t know) | Initiation of alcohol use by follow-up that parents did not know about (yes/no) | 17 months (range of 12-26 months) | Generalized Estimating Equations |
| Morgenstern *et al* (2014); Germany, Italy, Poland and Scotland; School | Mean = 13.5 years  Baseline: n=12464  Follow-up: n=10259  Binge subset: n=7438 | F=49.1%  M=50.9% | Family Affluence:  Low: 12.6%  Medium: 40.8%  High: 46.6% | Not reported | Longitudinal  **(Strong)** | Name of brand in favourite alcohol advertisement (receptive to alcohol marketing / not receptive to alcohol marketing) | Binge drinking (≥5 drinks in one occasion; yes/no) | 12 months | Logistic regression |
| Pasch *et al* (2009);  USA; Community and School | Mean:16.4  (n=242) | M=48.8% F=51.2% | Parent eductation: High school: 3.4% Some college: 18.4%  College degree: 33.1%  Training post college: 45.2% | 93.4% white | Cross-sectional **(Moderate)** | Outlet count, outlet density, distance to closest outlet; calculated as network and straight line buffer distances (3000m buffers); assessed for participant home and school. | No. of times drank alcohol in past month (ranging 0-40); no. of times got drunk in past month (ranging 0-≥10) | None | Linear regression |
| Paschall *et al* (2007); USA; School | 16-17  (n=3332) | M = 48.2%  F = 51.8% | Not reported | 84.5% white | Cross-sectional **(Weak)** | School district level alcohol sales across 3 months (low (0–17%), medium (20–38%), and high (40–100%) sales rates) | Past 30-day alcohol use (7-point scale converted to dichotomous measure of yes/no); heavy episodic drinking (≥5 drinks in a couple of hours; 7-point scale converted to dichotomous measure of yes/no) | None | Multi-level logistic regression |
| Rowland *et al* (2014); Australia; Community and School | 12-17  (n=10143) | %female: 48.1 | SEIFA: 1016.95 | Mother born in Australia: 63.32%  Father born in Australia: 60.21% | Cross-sectional **(Moderate)** | Outlet density count (outlets per 1,000 residents); organised into 4 categories: general density, packaged outlet density, on premise density, club density | Past 30-days alcohol use (yes/no); consumption of more than a few sips in past 12 months (never / ≥1 times) | None | Multi-level modelling |
| Saffer and Dave (2006); USA; Community and School | Data set 1 (MTF):  Mean: 15.731  (n=>63,000); Data set 2 (NLSY): Mean: 15.1186 (n=10,000) | Data set 1 (MTF):  M=48%  F = 52%  Data set 2 (NLSY):  M= 51.35%  F= 48.65% | Data set 1 (MTF):  Average weekly income in dollars: 27.6654  Data set 2 (NLSY):  Total past year income in dollars: 412.1189 | Data set 1 (MTF):  White: 64.45%  Black: 11.09%  Hispanic: 10.68%  Other: 13.78%  Data set 2 (NLSY):  White: 58.06%  Black: 16.23%  Hispanic: 12.82%  Other: 12.89% | Cross-sectional **(Weak)** | 1. Mean unit count of beer, wine and spirits advertising on TV, radio, outdoors, newspapers/magazines; liquor advertising outdoors and in newspapers/magazines in the respondent’s city of residence.  2.Weighted mean average price of alcohol adjusted by the inter-city cost of living index and the annual consumer price index. | Past month drinking (yes/no); past year drinking (yes/no); binge drinking (dataset 1: ≥5 drinks on at least one occasion in past two weeks; yes/no; dataset 2: ≥5 drinks on at least one occasion in past 30-days) | None | Panel regression |
| Shamblen *et al* (2011); USA; School and Community | 6^th^-8^th^ grade students  (n=5903) | %male: 48 | Not reported | White: 52%  Black: 16%  Hispanic: 27% | Longitudinal cohort **(Strong)** | Outlet density count per school census tract | Dichotomised measure of ever drank alcohol; any drinking in past year; any drinking in past 30-days (none/any alcohol use) | 2 years | Hierarchical linear modelling |
| Stacy *et al* (2004); USA; School | Mean: 12.5 at baseline  (n=2250) | %female: 51 | Not reported | Hispanic: 55%  Asian: 19%  Non-Hispanic White: 14%  African American: 2%  Pacific Islander: 1%  Native American: 1%  Multi-ethnic: 5%  Not reported: 3% | Longitudinal cohort **(Strong)** | 1. Self-reported exposure to TV alcohol advertising assessed using 2 items: 1. how often exposed to TV alcohol advertisements in past 6 months (7-point scale); 2. No. of alcohol advertisements exposed to in past week (7-point scale ranging 0-≥6 advertisements)  2. Exposure to advertising via popular TV shows assessed as frequency of watching 20 popular TV shows in past month (6-point scale ranging ‘never’ to ‘every day’).  3. Past month exposure to advertising via TV sports (6-point scale ranging ‘never’ to ‘every day’) | Current alcohol use: no. of days in the past 30-days had at least one drink of beer or wine/liquor (0/≥1); binge drinking (no. of times consumed ≥3 drinks within a few hours in past 30-days (0/≥1) | Collected at 2 time-points (7^th^ to 8^th^ grade) | Logistic regression |
| Stanley *et al* (2011); USA; Community | Grade 7-9: n=83,454  Grade 10-12: n=68,249 (n=151,703) | %female: 50 | Not reported | White: 62.7%  African American: 13.4%  Mexican-American: 14.2% | Cross-sectional **(Moderate)** | On and off premise liquor outlets count per 1,000 habitants (calculated separately and combined); no. of additional stores selling beer / wine. | No. of times drank alcohol in past month (0, 1-2, 3-9, 10-19, ≥20) | None | Multi-level modelling |
| Swahn *et al* (2011)  Zambia; School | 11-16  (n=371,194) | %male: 52.3 | Not reported | Not reported | Cross-sectional **(Weak)** | Exposure to billboard advertisements; provision of free drinks by an industry rep (any exposure / no exposure) | Lifetime drunkenness (no. of times gotten drunk on 4-item scale ranging 0-≥10 times; converted to dichotomous yes/no) | None | Logistic regression |
| Swahn *et al* (2013)  Philippines; School | 11-16yrs  (n=5290) | M=2279 (44%)  F=2986 (56%) | Not reported | Not reported | Cross-sectional **(Weak)** | Exposure to alcohol advertisements via: sports / public events, during sports on TV, billboards, newspapers / magazines, possessing alcohol brand logos and being offered alcohol from an alcohol company representative (any exposure / no exposure) | Current alcohol use (at least one drink ≥ days during past 30-days; yes/no); Lifetime drunkenness (no of times gotten drunk on 4-item scale ranging 0-≥10 times; converted to dichotomous yes/no) | None | Logistic regression |
| Tobler *et al* (2009a; 2009b; 2011); USA  School | 2009: 6– 8^th^ grade  (n=5,655)  2011: 6-8th grade; 12^th^ grade (n=4,027) | %male: 50 | 75% low income | African American: 59%  Hispanic: 41% | Longitudinal cohort **(Strong)** | Count of alcohol advertisements within 1,500 feet of study schools (average calculated); mean no. of off-sale alcohol outlets per 1,000 population per community area | No. of drinking occasions in past 12 months; drinking occasions in past 30-days; drinking occasions in past 7 days (response items included 0, 1-2, 3-5, 6-9, 10-19, 20-39, ≥40); binge drinking in past two weeks (≥5 drinks in a row; response items included never, once, twice, 3-5, 6-9, ≥10) | Collected at 4 TPs (beginning and end of 6^th^ grade followed by annual intervals) | Multilevel structural equation modelling |
| Truong and Sturm (2009); Truong (2008); USA; Community | 12-17  Mean: 14.3  (n=3660) | %female: 48.9% | Paid employment in past 12 months: 40.5%  Parents married or living with partner: 82.3% | Not reported | Cross-sectional **(Weak)** | On- and off- licence outlet density count (combined and separately): 1. close proximity: circular bands with a radii of 0,1 mile, 0.3 mile and 0.5 mile; 2. outlets beyond walking distance: circular bands 0.5-1.0 mile and 1.0-2.0 miles away from the respondents' residence. | At least 1 alcohol drink in past 30-days (yes/no); at least 1 heavy drinking episode in past 30-days (≥5 drinks in a row; yes/no) | None | Multivariate logistic regression |
| Van Hoof *et al* (2008); Netherlands; School | 14-17  (n=172) | M=52% (n=214)  F=48% (n=197) | Not reported | Not reported | Cross-sectional  **(Weak)** | Exposure to / use of alcohol price discounts in the past year (count) | Effect of exposure to / use of price discounts on alcohol consumption (5-point scale ranging ‘totally disagree’ to ‘totally agree’) | None | Descriptive statistics |
| Workman (2003);  USA; School | Mean=14.79  (n=262) | F=154  M=106  (2 missing data on gender) | Self-reported, ranging from 1 (lower-lower) to 9 (upper-upper); mean = 5 (middle-middle) | Caucasian: 205  Black:36  Other:21 | Cross-sectional **(Weak)** | Ownership of APCIs (yes/no; index also created by summing the items owned); exposure to APCIs at school (yes/no); use of ACPIs at school (yes/no) | Ever tried alcohol (yes/no); last time drank alcohol (past 7 days/past 30-days /1-6 months /more than 6 months ago); last time drank so much became drunk (past 7 days/past 30-days /1-6 months /more than 6 months ago); drinking frequency (at least once per day /at least once per week /sometimes /few times or less /no longer drink /never tried alcohol) | None | Descriptive statistics, t tests and chi-square tests. |
| Young *et al* (2013);  Scotland; School and Community | 15  (n=868) | M=432  F=436 | Missing: n=149  Manual: n=363  Non-manual: n=356 | Not reported | Cross-sectional **(Weak)** | Outlet density (total count per data zone with a mean population of 832); outlet proximity (distance in metres between home postcode and nearest outlet; 0-200 / 200.01-400 / 400.01-600 / 600.01-800 / ≥800.01); coded into 4 outlet categories (off-sales: 0-10 / 11-20 / 21-30 / ≥31; on-sales: 0-3 / 4-9 / 10-19 / ≥20) | Drinking frequency (weekly drinking / less frequent use) | None | Logistic regression |
| Zogg (2004); USA; School | 12-15  (n=1097) | M=539 (49%)  F=558 (51%) | Not reported | Latino/ Hispanic:60%  White:13%  Asian-American: 18% African-American: 1.5%  Almost 1% Native American, American Indian or Alaska Native; less than half of 1% Pacific Islander. | Prospective cohort **(Moderate)** | 1. Self-reported exposure to TV alcohol advertising using 2 items: 1. how often exposed to alcohol advertisements (5-point scale ranging ‘a lot’ to ‘never watch TV); 2. No. of alcohol advertisements exposed to in past week (7-point scale ranging 0-≥6 advertisements)  2. Exposure to advertising via popular TV shows assessed as frequency of watching 20 popular TV shows in past month (6-point scale ranging ‘never’ to ‘every day’).  3. Past month exposure to advertising via TV sports (6-point scale ranging ‘never’ to ‘every day’) | 8th grade alcohol use (3 indexes of beer, wine / liquor use; binge drinking calculated from alcohol use in the past 30-days, last 6 months and lifetime use) | 3-waves from 7th to 9th grade. | Multiple, hierarchical and stepwise regression |

**Table S2: Results summarised by behavioural outcome measure**

|  | **Drinking outcome** | | | |
| --- | --- | --- | --- | --- |
| **Study** | **Initiation (10 index studies)** | **Continuation (12 index studies)** | **Frequency (19 index studies)** | **Intensity (19 index studies)** |
| Azar *et al* (2016); **Place** | Not addressed | Not addressed | General, off-premises and on-premises density significantly associated with past month alcohol use among all adolescents (general AOR: 1.10 p<0.01 CI=1.07-1.14; on-premise AOR: 1.03 p<0.01 CI=1.02-1.05; off-premise AOR: 1.19 p<0.01 CI=1.11-1.28). | Significant association between off-premises outlet density and risky drinking among all adolescents in urban but not regional areas (AOR: 1.36 p<0.05 CI=1.05-1.75).  General and on-premises density significantly associated with risky drinking among all adolescents (general AOR: 1.10 p<0.01 CI=1.05-1.14; on-premise AOR: 1.05 p<0.01 CI=1.03-1.08) and current drinkers (general AOR: 1.07 p<0.01 CI=1.02-1.13; on-premise AOR: 1.04 p<0.01 CI=1.02-1.07)  Club density significantly associated with risky drinking among all students and past week drinkers in urban areas (all AOR: 1.94 p<0.01 CI=1.46-2.59; current AOR: 1.86 p<0.01 CI=1.32-2.63) |
| Bendtsen *et al* (2013); **Place** | Not addressed | Not addressed | Not addressed | No significant relationship between exposure to outlets near school and lifetime drunkenness: MODEL 1: OR=0.93, CI=0.67-1.29; MODEL 2: OR=0.95, CI=0.70-1.29. |
| Chen *et al* (2010); **Place** | Not addressed | Not addressed | Controlling for other factors, the initial level of drinking frequency was positively related to outlet density (coefficient=0.0003, p=0.045).  Controlling for other factors, growth of drinking frequency was negatively related to outlet density (coefficient=0.0002, p=0.028). The relationship between outlet density and drinking was mitigated by friends with access to a car.  Latino youths drank alcohol more frequently. They also tended to live in zip codes with higher alcohol outlet densities and lower median household income. The effect of Latino ethnicity may confound with the effects of zip code alcohol outlet density and median household income. Findings may simply reflect Latinos’ drinking and Latinos’ concentration in zip codes with high alcohol outlet density and low household income. | Controlling for other factors, the initial level of frequency of excessive drinking related positively to outlet density (coefficient=0.0009, p=0.000).  Controlling for other factors, growth of frequency of excessive drinking was related negatively to outlet density (coefficient=0.0004, p=0.008). The relationship between outlet density and drinking was mitigated by friends with access to a car. |
| Collins *et al* (2007); **Promotion** | Not addressed | Of 7 measures studied, ESPN beer adverts, other sports beer adverts, other TV beer adverts, beer concessions, in-store beer displays and API ownership all predicted grade 7 beer drinking (ESPN: OR=1.08, CI=.83-1.42; other sports: OR=1.19, CI=1.01-1.40; other TV: OR=1.13, CI=.95-1.34; beer concessions: OR=1.01, CI=.91-1.13; in-store displays: OR=1.03, CI=.92-1.14; API ownership: OR=1.76, CI=1.23-2.52). | Not addressed | Not addressed |
| de Bruijn *et al* (2012; 2013; 2016a; 2016b); **Promotion** | Not addressed | Not addressed | Exposure to TV advertising, online alcohol marketing and ABI ownership all significantly increased the odds of drinking in the past 30 days (TV: OR=1.21 p<.05 CI=0.97-1.50; ABIs: OR=1.29 p<.001 CI=1.17-1.42; online: OR=1.26 p<.001 CI= 1.17-1.36).  Online alcohol marketing at TP1 was positively related to alcohol use at TP2 (beta=.12, p<.001). The influence of online marketing was most pronounced when there was no alcohol use reported at TP1.  The effect of alcohol marketing exposure (measured using a 13-item latent variable) at TP1 to frequency of past-month drinking at TP2 was statistically significant [estimate=0.420; SE=0.058; CI=0.324 – 0.515; p<0.001, standardized estimate=0.140]. Alcohol marketing exposure at TP2 also had a significant effect on frequency of past-month drinking at TP3 (estimate=[.200; SE=0.044; CI=0.127 – 0.272; p<0.001, standardized estimate=0.084). | Exposure to TV advertising, online alcohol marketing and ABI ownership all significantly increased the odds of being a recent binge drinker (TV: OR=1.20 p<.05 CI=0.95-1.52; ABIs: OR=1.15 p<.001 CI=1.05-1.26; online: OR=1.24 p<.001 CI= 1.16-1.32). Dose-response relationship identified between online marketing and probability of recent binge drinking in all four countries (p<0.01)  The effect of alcohol marketing exposure (measured using a 13-item latent variable) at TP1 to frequency of past-month binge drinking at TP2 was statistically significant (estimate=0.409; SE=0.054; CI=0.320– 0.499; p<0.001, standardized estimate=0.142). Alcohol marketing exposure at TP2 also had a significant effect on frequency of past-month binge drinking at TP3 (estimate=0.168; SE=0.050; CI=0.086 – 0.250; p=0.001, standardized estimate =0.073). |
| Dumsha (2008; 2011); **Product** | Introduction of alcopops had no significant immediate or long-term effect on age at first drink.  Study results did not differ when stratified by age, gender or ethnicity. | Introduction of alcopops had no significant immediate or long-term effect on lifetime drinking or current use.  Study results did not differ when stratified by age, gender or ethnicity. | Not addressed. | Introduction of alcopops had no significant immediate or long-term effect on episodic heavy drinking.  Study results did not differ when stratified by age, gender or ethnicity. |
| Ellickson *et al* (2005); **Promotion** | Controlling for other variables, exposure to in-store displays was the only measure (of 4 studied) to significantly predict drinking initiation (OR=1.42; p<0.05). | Not addressed | Controlling for other variables, exposure to magazine alcohol advertising and beer concession stands at sports/music events were the only measures (of 4 studied) to significantly predict drinking frequency in baseline drinkers (magazines: coefficient=0.10; sport/music events: coefficient=0.09; p<0.05). | Not addressed |
| Faria *et al* (2011); **Product, promotion** | Not addressed | Not addressed | Past 30-day beer drinking associated with having a favourite brand (OR=5.150, CI=3.355-7.906, p<0.001); considering parties attended to be similar to those in advertisements (OR=1.712, CI=1.146-2.559, p=0.009); paying more attention to advertisements (OR=1.563, CI=1.048-2.330, p=0.028) and believing advertisements tell the truth (OR=2.122, CI=1.460-3.086, p<0.001). | Not addressed |
| Fisher *et al* (2007); **Promotion** | Owning/being willing to use APIs predictive of alcohol initiation among boys and girls (boys: OR=1.78, CI=1.36-2.33; girls: OR=1.74, CI=1.37-2.19). Effects greater among older (15+) boys (older: OR=2.43, CI=1.51-3.91; younger: OR=1.50, CI=1.08-2.09). | Not addressed | Not addressed | Owning/being willing to use APIs predicted binge drinking among girls (OR = 1.79, CI = 1.16-2.77).  Owning/being willing to use APIs did not predict binge drinking among boys (OR = 0.87, CI = 0.51-1.48). |
| Gordon *et al* (2010a; 2010b; 2011); **Promotion; Product** | Baseline marketing involvement significantly increased the odds of drinking initiation at follow-up (AOR=1.31, CI=1.003-1.711, p<0.05).  No association between drinking uptake at follow-up and awareness or no. of brands recalled at baseline. | Not addressed | Higher marketing involvement at baseline significantly increased the odds of fortnightly and monthly drinking at follow-up (fortnight: AOR=1.43, CI=1.146-1.795, p<0.01; monthly: AOR=1.33, CI=1.072-1.644, p<0.05). Uptake of fortnightly drinking at follow-up was also significantly associated with marketing awareness at baseline (AOR=1.11, CI=1.005-1.234, p<0.05).  No association between uptake of fortnightly drinking at follow-up and no. of brands recalled at baseline. No association between uptake of monthly drinking at follow-up and awareness of alcohol marketing or no. of brands recalled at baseline. | Not addressed |
| Grenard *et al* (2008; 2013); **Promotion** | Not addressed | Not addressed | An interaction between exposure to advertisements and liking of advertisements in 7th grade was predictive of past 30-days and past 6-months alcohol use (SHOWS X LIKING: girls: 0.091, SE=0.042, p<.05; boys: 0.093, SE=0.046, p<.05). Frequency of watching popular shows at TP1 predicted growth in alcohol use for girls only (0.190, SE=0.058, p<.01). | Not addressed |
| Henriksen *et al* (2008); **Promotion; Product** | Never drinkers who reported high or moderate baseline alcohol marketing receptivity were more likely to initiate drinking by follow-up. Better brand recall at baseline was also associated with increased odds of initiating drinking by follow-up (high receptivity: OR=1.68, CI=1.20-2.35; moderate receptivity: OR=1.20, CI=.75-1.90; brand recall: OR=1.10, CI=.97-1.25). | Current alcohol use at follow-up was more likely among those who reported high or moderate baseline alcohol marketing receptivity. Smaller increases in the odds of alcohol use at follow-up were also associated with better brand recall at baseline (high receptivity: OR=1.62, CI=1.01-2.60; moderate receptivity: OR=1.19, CI=.62-2.26; brand recall: OR=1.13, CI=.94-1.33). | Not addressed | Not addressed |
| Huckle *et al* (2008); **Place** | Not addressed | Not addressed | Annual drinking frequency not predicted by outlet density (beta=-0.001, t-stat=-0.333, p-value – non-significant). | Typical-occasion quantity consumption predicted by outlet density (beta = 0.004, t-stat = 2.000, p<0.05).  Outlet density (beta=0.005, t-value=1.666, p=0.058) approached significance only as predictor of frequency of drunkenness. |
| Jones and Magee (2011); **Promotion** | Controlling for other variables, exposure to advertising via magazines, bottleshops, bar/pubs or promotional materials were the only measures (of 7 studied) that predicted drinking initiation (magazine: AOR=1.69, CI=1.20-2.38; bottleshop: AOR=1.49, CI=1.04-2.14; bar/pub: AOR=1.49, CI=1.10-2.01; promo material: AOR=1.36, CI= 1.01-1.84).  No significant association between drinking initiation and exposure to alcohol media of any type for males and females aged 12-15 and males aged 16-17. | Not addressed | Controlling for other variables, exposure to advertising in a pub/bar was the only measure (of 7 studied) that predicted past 12-month alcohol use (AOR=1.69, CI=1.27-2.25).  Exposure to TV advertisements was associated with reduced odds of past 12-month alcohol use (AOR=0.55, CI=0.30-0.99).  Exposure to advertising via magazines, internet or in a pub/bar were the only measures (of 7 studied) that predicted past 4-week alcohol use (magazine: AOR=1.54, CI=1.11-2.14; internet: AOR=1.36, CI=1.03-1.79; pub/bar: AOR=1.44, CI = 1.09-1.91).  No significant association between past 12-month alcohol use and exposure to alcohol media of any type among females aged 16-17. No significant association between past 4-week alcohol use and exposure to alcohol media of any type among females. | Not addressed |
| Kuntsche *et al* (2008); **Place** | Not addressed | Not addressed | On-premise outlet density positively related to QF (beta=0.19, t-value=2.1, p<0.05). | On- and off-premises outlet density not positively related to RSOD. |
| Lin *et al* (2012); **Product; promotion** | Not addressed | Having a favourite brand increased the odds of being a drinker (OR=4.56, CI=3.62-5.76).  Awareness of each alcohol marketing channel and engagement with traditional marketing increased probability of drinking (awareness: OR=1.08, CI=1.03-1.13; engagement: OR =1.51, CI=1.19-1.93).  Engagement with web-based marketing also increased probability of drinking (OR=1.98, CI=1.22-3.24). Effects greater for those who engaged in both traditional and web-based marketing (OR=2.25, CI=1.57-3.22). | Having a favourite brand increased frequency of alcohol consumption (OR=1.65, CI=1.41-1.92).  Engagement with both traditional and web-based alcohol marketing also increased frequency of alcohol consumption (OR=1.34, CI=1.08-1.66). | Having a favourite alcohol brand increased drinking amount on a typical drinking occasion (OR=1.86, CI=1.57-2.21). |
| Lo *et al* (2013a; 2013b); **Place** | Not addressed | Not addressed | Outlet density had no significant impact on 30-day alcohol use in urban or rural environments (urban: Coeff=-.002, SE=.002; Coeff=-.001, SE=.002; rural: coeff=-.014, SE=.024; coeff=-.009, SE=.024; spatial lag: urban: coeff=.000, SE=.004; rural: coeff=-.012, SE=.039) | Outlet density significantly associated with reduced binge drinking (-.002, p<.05).  Binge drinking increased with grade level. This association became stronger among students living in neighbourhoods with high outlet density (.003, p<.05). |
| McClure *et al* (2006); **Promotion** | ABM owners at follow-up more likely to have initiated drinking than non-owners (CI=1.1-2.0, p<0.001). Controlling for covariates, this relationship was significant for females only (OR=3.33, CI=1.7-6.3, p=0.02). | Not addressed | Not addressed | Not addressed |
| McClure *et al* (2009; 2013); Stoolmiller *et al* (2012); **Promotion** | ABM owners at TP2 (8 months) more likely to have initiated alcohol use by TP3 (16 months) (HR=1.66, CI=1.15-2.40). Non-owners who had initiated drinking at TP2 were also more likely to own ABM by TP3 (HR=1.41, CI=1.09-1.83). ABM owners at TP2 were more likely to have initiated drinking at TP4 (24 months) (AHR=1.44 CI=1.19-1.74). | Not addressed | Not addressed | ABM ownership had a direct effect on binge drinking initiation 16-24 months later (HR=2.22, CI=1.49-3.32). ABM owners at TP2 (8 months) were also more likely to initiate binge drinking at T4 (24 months) (AHR=1.24, CI=1.00-1.54).  ABM ownership and having a favourite alcohol brand or advertisement were significantly associated with binge drinking (ABM: p<0.0001; favourite advertisement: p=0.001; favourite brand: p<0.0001). However, only ABM ownership had an independent association with binge drinking, indicating a direct pathway (0.24, SE=0.06, p<0.001). |
| Morgenstern *et al* (2014); **Promotion** | Having a favourite advertisement was significantly associated with drinking initiation in baseline never drinkers (AOR=1.45 CI=1.26-1.66). | Not addressed | Not addressed | Having a favourite advertisement was significantly associated with binge drinking (AOR=2.13 CI=1.92-2.36) |
| Pasch *et al* (2009); **Place** | Not addressed | Not addressed | HOME: Distance/density/count of outlets on either a network or straight line route not related to past-month alcohol use (density (network): -1.47, SE=12.34, p=0.91; density (straight line): 2.85, SE=15.32, p=0.85; count (network): 0.003, SE=0.01, p=0.77; count (straight line): 0.001, SE=0.01, p=0.81; distance (network): -0.00002, SE=0.00, p=0.17; distance (straight): -0.00003, SE=0.00, p=0.19)  SCHOOL: Distance/density/count of outlets on either a network or straight line route not related to past-month alcohol use (density (network): -2.03, SE=15.56, p=0.90; density (straight line): 1.60, SE=17.80, p=0.93; count (network): -0.002, SE=0.01, p=0.87; count (straight line): 0.0003, SE=0.01, p=0.97; distance (network): -0.00004, SE=0.00, p=0.14; distance (straight): -0.00004, SE=0.00, p=0.23) | HOME: Distance / density / count of outlets on either a network or straight line route was not related to past month drunkenness (density (network): 7.30, SE=8.39, p=0.39; density (straight line): 13.30, SE=10.40, p=0.20; count (network): 0.008, SE=0.01, p=0.22; count (straight line): 0.005, SE=0.00, p=0.20; distance (network): -0.00001, SE=0.00, p=0.17; distance (straight): -0.00002, SE=0.00, p=0.15).  SCHOOL: Distance / density / count of outlets on either a network or straight line route was not related to past month drunkenness (density (network): 0.83, SE=10.46, p=0.94; density (straight line): -0.55, SE=11.96, p=0.96; count (network): -0.001, SE=0.01, p=0.87; count (straight line): -0.001, SE=0.00, p=0.85; distance (network): -0.00003, SE=0.00, p=0.19; distance (straight): -0.00002, SE=0.00, p=0.35) |
| Paschall *et al* (2007); **Place** | Not addressed | Not addressed | Use of commercial and social alcohol sources positively related to past 30-day alcohol use (commercial OR=5.93, CI=2.99-11.74, p<0.001; social OR=139.95, CI=104.47-187.49, p<0.001).  ORs for use of social sources considerably larger than ORs for use of commercial alcohol sources. | Not addressed |
| Rowland *et al* (2014); **Place** | Not addressed | Overall and individually outlet types were significantly associated with alcohol use only when included as an interaction with age (overall: OR=1.002, p=.001; package: OR=1.035, p=.000; general: OR=1.007, p=.002; on premise: OR = 1.003, p=.001; club: OR=1.035, p=.021).  The largest proportional change arose for packaged density; the smallest was for on-premise density and general density. For each type of outlet effects were greatest for younger individuals (PACKAGE: 12: percentage increase (PI) per 10% increase in density=5.30, CI=1.83-8.75; 13: PI=4.36, CI=1.31-7.42; 14: PI=3.03, CI=0.26-5.80; 15: PI=0.96, CI=-2.14-4.07; 16: PI=-2.66, CI=-7.82-2.49; 17: PI=-1.07, CI=-22.88-1.55; GENERAL: 12: PI=1.31, CI=0.40-2.24; 13: PI=1.08, CI=0.28-1.89; 14: PI=0.74, CI=0.03-1.45; 15: PI=0.22, CI=-0.54-0.97; 16: PI=-0.71, CI=-1.96-0.55; 17: PI=-2.74, CI=-5.81-0.32; ON PREMISE: 12: PI=1.68, CI=0.59-27.96; 13: PI=1.38, CI=0.42-23.36; 14: PI=0.92, CI=0.01-1.75; 15: PI=0.22, CI=-0.64-1.07; 16: PI=-1.03, CI=-2.48-0.42; 17: PI=-3.85, CI=-7.71-0.00; CLUB: 12: PI=2.80, CI=0.49-5.12; 13: PI=2.45, CI=0.24-4.66; 14: PI=1.94, CI=-0.32-4.21; 15: PI=1.16, CI=-1.60-3.92; 16: PI=-0.19, CI=-4.41-4.02; PI: OR=-0.31, CI=-1.14-5.07).  Increased overall outlet density was significantly associated with an increase in alcohol use among 12-14 year-olds (12: PI=2.04, CI=0.74-3.35; 13: PI=1.66, CI=0.53-2.80; 14: PI=1.12, CI=0.14-2.11).  No relationship between overall outlet density and alcohol use among 15-17 year-olds (15: PI=0.29, CI=-7.32-1.31; 16: PI=-1.19, CI=-2.91-5.29; 17: PI=-4.452, CI=-9.04-0.01). | Not addressed | Not addressed |
| Saffer and Dave (2006); **Price,**  **Promotion** | Not addressed | Not addressed | Annual and monthly drinking reduced as price increased. Data set 1 (MTF**):** Annual drinking price elasticity: -0.1902, SE=0.0231; past month drinking price elasticity: -0.2639, SE=0.0370). Effects were larger for females and white young people (female: annual -0.2375, SE=0.0312; monthly -0.3956, SE=0.0513; white: annual-0.3053, SE=0.0274; monthly -0.4638, SE=0.0436). Data set 2 (NLSY): Past month drinking price elasticity: -0.4229, SE=0.2779).  Annual and monthly drinking increased as advertising increased. Data set 1 (MTF**):** Annual drinking advertising elasticity: 0.0173, SE=0.0038; past month drinking advertising elasticity: 0.0238, SE=0.0059). Effects were larger for females and white young people (females: annual 0.0195, SE=0.0050; monthly 0.0348, SE=0.0079; white: annual 0.0165, SE=0.0044; monthly 0.0207, SE=0.0069). Data set 2 (NLSY): Advertising exposure sig in 2 of 4 regressions exploring past month drinking (specification 2: 0.1627, Z score=2.12; specification 3: 0.2463, Z score=2.19). | Binge drinking reduced as price increased. Data set 1 (MTF): Binge drinking price elasticity: -0.1842, SE=0.0562. Effects were larger for females and white young people (female: -0.2369, SE=0.0803; white: -0.3611, SE=0.0658). Data set 2 (NLSY): Binge drinking price elasticity: -0.7307, SE=0.4897).  Binge drinking increased as advertising increased. Data set 1 (MTF): Binge drinking advertising elasticity: 0.0265, SE=0.0089). Effects were larger for females and white young people (females: 0.0280, SE=0.0122; white: 0.0213, SE=0.0103). Data set 2 (NLSY): Advertising exposure sig in 2 of 4 regressions exploring past month binge drinking (specification 1: 0.0809, Z score = 2.12; specification 2: 0.1441, Z score = 2.80). |
| Shamblen *et al* (2011); **Place** | Not addressed | Students in high off-trade outlet density communities increased their alcohol use between 6^th^ and 8^th^ grade; students attending schools in low outlet density communities had higher initial levels of alcohol use that remained relatively stable over time (lifetime: OR=.87; past year: OR=.88; past 30-days: OR=.88). | Not addressed | Not addressed |
| Stacy *et al* (2004); **Promotion** | Not addressed | Of 3 measures studied, adjusted for covariates,8^th^ grade beer and wine/liquor use were significantly associated with 7^th^ grade exposure to advertising via popular TV shows only (AOR=1.44, CI=1.27-1.61, p<.001). | Not addressed | Adjusted for covariates, 8^th^ grade binge drinking was not significantly associated with any measure of 7^th^ grade advertising exposure. |
| Stanley *et al* (2011); **Place** | Not addressed | Not addressed | Increase in per capita liquor outlets significantly (but weakly) associated with an increase in past month alcohol use for 7-9th grade students (OR=1.03, p<.01).  No significant associations found for 10-12th grade students.  An increase in the no. of per capita liquor outlets in ethnic communities was significantly associated with a decrease in past month alcohol use by adolescents in those communities compared with otherwise similar youth in White communities (African-American, 7-9th grade: OR=.90, p<.05; Mexican-American, 7-9th grade: OR=.83, p<.01; Mexican-American, 10-12th grade: OR=.74, p<.01). | Not addressed |
| Swahn *et al* (2011); **Promotion** | Not addressed | Provision of free alcohol from an alcohol industry rep associated with greater odds of current alcohol use (OR=4.37 CI=3.21-5.95).  Exposure to billboard advertisements associated with decreased odds of current alcohol use (OR=0.65 CI=0.46-0.92). | Not addressed | Provision of free alcohol from an alcohol industry rep associated with greater odds of drunkenness (OR=3.02 CI=2.34-3.90).  Exposure to billboard advertisements associated with decreased odds of drunkenness (OR=0.74 CI=0.56-0.98). |
| Swahn *et al* (2013); **Promotion** | Not addressed | Of 5 measures studied, exposure to alcohol marketing via brand logos and billboards; provision of alcohol via an industry rep and owning a gift with a brand logo were sig associated with increased current alcohol use (brand logo: OR=1.52, CI=1.26-1.84; billboards: OR= 1.32, CI=1.09-1.59; provision of free alcohol: OR=2.22, CI=1.57-3.13; gift with brand logo: OR=1.86, CI=1.53-2.27). | Not addressed | Of 5 measures studied, exposure to alcohol marketing via public advertisements and newspapers/magazines; and provision of free alcohol were associated with drunkenness (public ads: OR=1.50, CI=1.06-2.12; newspaper/magazines: OR=1.65, CI=1.05-2.58, provision of free alcohol: OR=1.84, CI=1.06-3.21). |
| Tobler *et al* (2009a; 2009b; 2011); **Place; promotion** | Not addressed | Exposure to alcohol advertisements significantly associated with alcohol use in 8^th^ grade (b= 0.049, *p* < 0.05).  Outlet density did not have a significant direct effect on alcohol use in 8th grade.  Among low income African American adolescents, the effects of alcohol outlet density at baseline on alcohol use in 12th grade were mediated entirely by beliefs favourable to use (beta=.037, p=.001) and deviant peer affiliations (beta=.016, p=.017).  Among low income Hispanic adolescents, the effects of alcohol advertisement exposure on alcohol use in 12th grade were entirely mediated through beliefs favourable to use in 8th grade (beta=.014, p=.076). | Not addressed | Not addressed |
| Truong and Sturm (2009);  Truong (2008); **Place** | Not addressed | Not addressed | No association between past 30-day drinking and on- or off-sales outlets within 0.5 miles (on-sales: OR = 1.01, CI=0.99-1.03; off-sales: OR=1.00, CI=0.94-1.07).  No association between past 30-day drinking and outlets located over 0.5 miles away (off-sales 0.5-1.0 mile: OR=0.98, CI=0.94-1.02; off-sales 1.0-2.0 miles: OR=1.00, CI=0.98-1.01; on-sales 0.5-1.0 miles: OR=1.00, CI=0.98-1.02; on-sales 1.0-2.0 miles: OR=1.00, CI=1.00-1.01). | Total no. of alcohol outlets within 0.5 miles was significantly associated with past 30-day binge drinking (overall: OR=1.03, CI=1.01-1.05, p<0.01; on-sales: OR=1.03, CI=1.01-1.07, p<0.05; off-sales: OR=1.03, CI=1.01-1.07, p<0.05).  Outlets located over 0.5 miles away had no relationship with past 30-day binge drinking.  The number of alcohol outlets was significantly higher around residences of minority and lower-income families. In turn, binge drinking among adolescents aged 12 to 17 years were significantly associated with the presence of alcohol retailers within 0.5 miles of home. Simulation of changes in the alcohol environment showed that if alcohol sales were reduced from the mean number of alcohol outlets around the lowest-income quartile of households to that of the highest quartile, prevalence of binge drinking would fall from 6.4% to 5.6%. |
| Van Hoof *et al* (2008); **Price** | Not addressed | Alcohol discounts had a significant effect on alcohol consumption (m=3.39, SD=0.76, t[149]=6.25, p=0.000). This effect did not sig differ between age groups (14-15 and 16-17) (t[138,54]=0.91, p=0.367) | Not addressed | Not addressed |
| Workman (2003); **Promotion** | Non-APCI owners were more likely to have never tried alcohol or be occasional drinkers only (never tried: n=59, 35.5% versus n=13, 13.7%; occasional: n=65, 39.2% versus n=23, 24.2%). Non-owners were also more likely to indicate that they do not drink and never will compared to APCI owners (n=48, 29.1% versus n=13, 13.5%). |  | Those who reported seeing an APCI were more likely to drink at least once per week (but not every day) or ‘sometimes’ (less than once per week) compared to those who did not report seeing an APCI (at least once per week: n=17, 12.7% versus n=6, 4.7%; sometimes: n=44, 32.8% versus n=23, 18.1%).  APCI owners were more likely to drink at least once per week (but not every day) and ‘sometimes’ (less than once per week) compared to non-owners (at least once per week: n=16, 16.8% versus n=7, 4.2%; sometimes: n=36, 37.9% versus n=31, 18.7%). Non-owners were more likely to have never tried alcohol or be occasional drinkers only (never tried: n=59, 35.5% versus n=13, 13.7%; occasional: n=65, 39.2% versus n=23, 24.2%). | Not addressed |
| Young *et al* (2013); **Place** | Not addressed | Not addressed | Significant associations found between alcohol use and highest no. of off-sale outlets within 1200m (unadjusted for sample clustering: OR=1.60, CI=1.09-2.36, p=0.016; AOR=1.49, CI=1.00-2.21, p=0.046; adjusted for sample clustering: OR=1.54, CI=1.02-2.32, p=0.041).  Significant associations found between alcohol use and distance to nearest off-sales outlet (200.01-400m: unadjusted for sample clustering: OR=1.55, CI=1.02-2.36, p=0.041; 0-200m: unadjusted for sample clustering: OR=1.97, CI=1.25-3.11, p=0.004; AOR=2.00, CI=1.25-3.20, p=0.004, adjusted for sample clustering: OR=1.93, CI=1.21-3.08, p=0.006; AOR=1.98, CI=1.23-3.19, p=0.005). | Not addressed |
| Zogg (2004); **Promotion** | Not addressed | Exposure to TV advertisements at TP1 significantly predicted TP2 beer use (.058, p<.05).  Exposure to TV advertisements at TP1 did not predict wine/liquor use at TP2.  Exposure to advertising via popular TV shows significantly predicted beer and wine/liquor use for white respondents only at TP2 (beer: .278, p<.001; wine/liquor: .309, p<.001).  Exposure to advertising via TV sports significantly predicted beer and wine/liquor use for white respondents only at TP2 (beer: .240, p<.01; wine/liquor: .174, p<.05). | Not addressed | Exposure to TV advertisements at TP1 did not predict binge drinking at TP2.  Exposure to advertising via popular TV shows significantly predicted binge drinking for white respondents only (.354, p<.05).  Exposure to advertising via TV sports significantly predicted binge drinking for white respondents only (.526, p<.01). |

**Table 3: Promotional studies with a binge drinking (5+drinks) outcome measure (n=7 studies)**

| **Study and setting** | **Age (years)**  **Sample** | **Study design;**  **Quality rating** | **Exposure Measure(s)** | **Behavioural Outcome Measure(s)** | **Follow up (rate / duration)** | **Reported analyses** | **Results** |
| --- | --- | --- | --- | --- | --- | --- | --- |
| de Bruijn *et al* (2012; 2013; 2016a; 2016b); Germany; Italy; Netherlands; Poland  School  **Promotion** | TP1: mean: 14.05yrs  (n=9032)  TP1 and TP2: mean: 13.95yrs (n=6651)  TP3: mean: 14yrs (n=9075) | Longitudinal; collected at three TPs **(Strong)** | 1. Frequency of exposure to alcohol marketing in online media (never / rarely / sometimes / often / very often; categorized 1-5)  2. Ownership of ABIs (yes/no)  3. Frequency of exposure to TV alcohol advertising, measured as frequency of viewing 8 selected TV programmes (total score of between 0 and 1 calculated for each respondent)  4. 13-item latent variable measuring exposure to online alcohol marketing, televised alcohol marketing, alcohol sport sponsorship, music event / festival sponsorship, ownership of ABIs, reception of free samples and exposure to price offers. | TP1: Onset of binge drinking (≥5 drinks in a single occasion in last 30 days; yes/no)  TP1, TP2 and TP3: Frequency of binge drinking (≥5 drinks in a single occasion) in last 30 days (0; 1; 2; 3-5; 6-9; ≥10) | Three waves (14-17 month period between TP1 and TP3) | TP1: Binary and logistic regression  TP1 and TP2: hierarchical regression  TP1, TP2 and TP3: auto-regressive cross-lagged modelling | Exposure to TV advertising, online alcohol marketing and ABI ownership all significantly increased the odds of being a recent binge drinker (TV: OR=1.20 p<.05 CI=0.95-1.52; ABIs: OR=1.15 p<.001 CI=1.05-1.26; online: OR=1.24 p<.001 CI= 1.16-1.32). Dose-response relationship identified between online marketing and probability of recent binge drinking in all four countries (p<0.01).  The effect of alcohol marketing exposure (measured using a 13-item latent variable) at TP1 to frequency of past-month binge drinking at TP2 was statistically significant (estimate=0.409; SE=0.054; CI=0.320– 0.499; p<0.001, standardized estimate=0.142). Alcohol marketing exposure at TP2 also had a significant effect on frequency of past-month binge drinking at TP3 (estimate=0.168; SE=0.050; CI=0.086 – 0.250; p=0.001, standardized estimate =0.073). |
| Fisher *et al* (2007)  USA; School  **Promotion** | 11-18  (n=5511) | Prospective cohort **(Strong)** | Awareness of alcohol advertising (yes/no); owning / being willing to use APIs (yes/no) | Binge drinking in past year (≥5 drinks within a few hours; yes/no) | 12 months | Logistic regression | Owning/being willing to use APIs predicted binge drinking among girls (OR = 1.79, CI = 1.16-2.77).  Owning/being willing to use APIs did not predict binge drinking among boys (OR = 0.87, CI = 0.51-1.48). |
| McClure *et al* (2009; 2013); Stoolmiller *et al* (2012)  USA; Telephone  **Promotion** | 10-14 at baseline  n=6552 (baseline)  n=5503 (8mths)  n=5019 (16mths)  n=4575 (24mths)  n=1734 (2013) | Longitudinal cohort; cross-sectional analysis at 6^th^ wave (2013) **(Moderate)** | Ownership of ABM at 8, 16 and 24 months (yes/no); favourite advertisement (yes/no; 6^th^ wave data only) | Binge drinking (≥5 drinks in a row within a couple of hours; yes/no); 30-day binge drinking (yes/no) | 4 waves over 24 months; cross-sectional analysis of wave 6 data | Panel and hazard logistic regression | ABM ownership had a direct effect on binge drinking initiation 16-24 months later (HR=2.22, CI=1.49-3.32). ABM owners at TP2 (8 months) were also more likely to initiate binge drinking at T4 (24 months) (AHR=1.24, CI=1.00-1.54).  ABM ownership and having a favourite alcohol brand or advertisement were significantly associated with binge drinking (ABM: p<0.0001; favourite advertisement: p=0.001; favourite brand: p<0.0001). However, only ABM ownership had an independent association with binge drinking, indicating a direct pathway (0.24, SE=0.06, p<0.001). |
| Morgenstern *et al* (2014)  Germany, Italy, Poland and Scotland; School  **Promotion** | Mean = 13.5 years  Baseline: n=12464  Follow-up: n=10259  Binge subset: n=7438 | Longitudinal  **(Strong)** | Name of brand in favourite alcohol advertisement (receptive to alcohol marketing / not receptive to alcohol marketing) | Binge drinking (≥5+ drinks in one occasion; yes/no) | 12 months | Logistic regression | Having a favourite advertisement was significantly associated with binge drinking (AOR=2.13 CI=1.92-2.36) |
| Saffer and Dave (2006)  USA; Community and School  **Promotion** | Data set 1 (MTF):  Mean: 15.731  (n=>63,000)  Data set 2 (NLSY):  12-16  Mean: 15.1186  (n=10,000) | Cross-sectional **(Weak)** | 1. Mean unit count of beer, wine and spirits advertising on TV, radio, outdoors, newspapers/magazines; liquor advertising outdoors and in newspapers/magazines in the respondent’s city of residence.  2.Weighted mean average price of alcohol adjusted by the inter-city cost of living index and the annual consumer price index. | Binge drinking (dataset 1: ≥5 drinks on at least one occasion in past two weeks; yes/no; dataset 2: ≥5 drinks on at least one occasion in past 30-days) | None | Panel regression | Binge drinking reduced as price increased. Data set 1 (MTF): Binge drinking price elasticity: -0.1842, SE=0.0562. Effects were larger for females and white young people (female: -0.2369, SE=0.0803; white: -0.3611, SE=0.0658). Data set 2 (NLSY): Binge drinking price elasticity: -0.7307, SE=0.4897).  Binge drinking increased as advertising increased. Data set 1 (MTF): Binge drinking advertising elasticity: 0.0265, SE=0.0089). Effects were larger for females and white young people (females: 0.0280, SE=0.0122; white: 0.0213, SE=0.0103). Data set 2 (NLSY): Advertising exposure sig in 2 of 4 regressions exploring past month binge drinking (specification 1: 0.0809, Z score = 2.12; specification 2: 0.1441, Z score = 2.80). |
| Stacy *et al* (2004)  USA; School  **Promotion** | Mean: 12.5 at baseline  (n=2250) | Longitudinal cohort **(Strong)** | 1. Self-reported exposure to TV alcohol advertising assessed using 2 items: 1. how often exposed to TV alcohol advertisements in past 6 months (7-point scale); 2. No. of alcohol advertisements exposed to in past week (7-point scale ranging 0-≥6 advertisements)  2. Exposure to advertising via popular TV shows assessed as frequency of watching 20 popular TV shows in past month (6-point scale ranging ‘never’ to ‘every day’).  3. Past month exposure to advertising via TV sports (6-point scale ranging ‘never’ to ‘every day’) | Binge drinking (no. of times consumed ≥3 drinks within a few hours in past 30-days (0/≥1) | Collected at 2 TPs (7^th^ to 8^th^ grade) | Logistic regression | Adjusted for covariates, 8^th^ grade binge drinking was not significantly associated with any measure of 7^th^ grade advertising exposure. |
| Zogg (2004)  USA; School  **Promotion** | 12-15  (n=1097) | Prospective cohort  **(Moderate)** | 1. Self-reported exposure to TV alcohol advertising assessed using 2 items: 1. how often exposed to TV alcohol advertisements in past 6 months (7-point scale); 2. No. of alcohol advertisements exposed to in past week (7-point scale ranging 0-≥6 advertisements)  2. Exposure to advertising via popular TV shows assessed as frequency of watching 20 popular TV shows in past month (6-point scale ranging ‘never’ to ‘every day’).  3. Past month exposure to advertising via TV sports (6-point scale ranging ‘never’ to ‘every day’) | Binge drinking calculated from alcohol use in the past 30-days, last 6 months and lifetime use) | 3-waves from 7th to 9th grade. | Multiple, hierarchical and stepwise regression | Exposure to TV advertisements at TP1 did not predict binge drinking at TP2.  Exposure to advertising via popular TV shows significantly predicted binge drinking for white respondents only (.354, p<.05).  Exposure to advertising via TV sports significantly predicted binge drinking for white respondents only (.526, p<.01). |
